# Multiple models for outbreak decision support in the face of uncertainty

**DOI:** 10.1073/pnas.2207537120

**Published:** 2023-04-25

**Authors:** Katriona Shea, Rebecca K. Borchering, William J. M. Probert, Emily Howerton, Tiffany L. Bogich, Shou-Li Li, Willem G. van Panhuis, Cecile Viboud, Ricardo Aguás, Artur A. Belov, Sanjana H. Bhargava, Sean M. Cavany, Joshua C. Chang, Cynthia Chen, Jinghui Chen, Shi Chen, YangQuan Chen, Lauren M. Childs, Carson C. Chow, Isabel Crooker, Sara Y. Del Valle, Guido España, Geoffrey Fairchild, Richard C. Gerkin, Timothy C. Germann, Quanquan Gu, Xiangyang Guan, Lihong Guo, Gregory R. Hart, Thomas J. Hladish, Nathaniel Hupert, Daniel Janies, Cliff C. Kerr, Daniel J. Klein, Eili Y. Klein, Gary Lin, Carrie Manore, Lauren Ancel Meyers, John E. Mittler, Kunpeng Mu, Rafael C. Núñez, Rachel J. Oidtman, Remy Pasco, Ana Pastore y Piontti, Rajib Paul, Carl A. B. Pearson, Dianela R. Perdomo, T. Alex Perkins, Kelly Pierce, Alexander N. Pillai, Rosalyn Cherie Rael, Katherine Rosenfeld, Chrysm Watson Ross, Julie A. Spencer, Arlin B. Stoltzfus, Kok Ben Toh, Shashaank Vattikuti, Alessandro Vespignani, Lingxiao Wang, Lisa J. White, Pan Xu, Yupeng Yang, Osman N. Yogurtcu, Weitong Zhang, Yanting Zhao, Difan Zou, Matthew J. Ferrari, David Pannell, Michael J. Tildesley, Jack Seifarth, Elyse Johnson, Matthew Biggerstaff, Michael A. Johansson, Rachel B. Slayton, John D. Levander, Jeff Stazer, Jessica Kerr, Michael C. Runge

**Affiliations:** ^a^Department of Biology, The Pennsylvania State University, University Park, PA 16802; ^b^Center for Infectious Disease Dynamics, The Pennsylvania State University, University Park, PA 16802; ^c^Nuffield Department of Medicine, Big Data Institute, Li Ka Shing Centre for Health Information and Discovery, University of Oxford, Oxford OX3 7LF, United Kingdom; ^d^State Key Laboratory of Grassland Agro-ecosystems, Center for Grassland Microbiome, and College of Pastoral, Agriculture Science and Technology, Lanzhou University, Lanzhou, 73000, People’s Republic of China; ^e^Department of Epidemiology, Graduate School of Public Health, University of Pittsburgh, Pittsburgh, PA 15260; ^f^Fogarty International Center, National Institutes of Health, Bethesda, MD 20892; ^g^Office of Biostatistics and Pharmacovigilance, Center for Biologics Evaluation and Research, Food and Drug Administration, Silver Spring, MD 20993; ^h^Department of Biology, University of Florida, Gainesville, FL 32611; ^i^Department of Biological Sciences, University of Notre Dame, Notre Dame, IN 46556; ^j^Epidemiology and Biostatistics Section, Rehabilitation Medicine, Clinical Center, National Institutes of Health, Bethesda, MD 20892; ^k^Mederrata Research Inc, Columbus, OH 43212; ^l^Department of Civil and Environmental Engineering, University of Washington, Seattle, WA 98195; ^m^Department of Computer Science, University of California, Los Angeles, Los Angeles, CA 90095; ^n^Department of Public Health Sciences, University of North Carolina at Charlotte, Charlotte, NC 28223; ^o^School of Data Science, University of North Carolina at Charlotte, Charlotte, NC 28223; ^p^Mechatronics, Embedded Systems and Automation Laboratory, School of Engineering, University of California, Merced, CA 95343; ^q^Department of Mathematics, Virginia Tech, Blacksburg, VA 24061; ^r^Mathematical Biology Section, Laboratory of Biological Modeling, National Institute of Diabetes and Digestive and Kidney Diseases, National Institutes of Health, Bethesda, MD 20892; ^s^Los Alamos National Laboratory, Los Alamos, NM 87545; ^t^School of Life Sciences, Arizona State University, Tempe, AZ 85287; ^u^School of Mathematics, Jilin University, Changchun, Jilin 130012, People’s Republic of China; ^v^Institute for Disease Modeling, Global Health Division, Bill & Melinda Gates Foundation, Seattle, WA 98109; ^w^Emerging Pathogens Institute, University of Florida, Gainesville, FL 32610; ^x^Department of Population Health Sciences, Division of Epidemiology, Weill Cornell Medicine, Cornell University, New York, NY 10065; ^y^Computational Intelligence to Predict Health and Environmental Risks, University of North Carolina at Charlotte, Charlotte, NC 28223; ^z^Department of Emergency Medicine, Johns Hopkins University, Baltimore, MD 21209; ^aa^One Health Trust, Washington, DC 20015; ^bb^Department of Integrative Biology, The University of Texas at Austin, Austin, TX 78712; ^cc^Department of Microbiology, School of Medicine, University of Washington, Seattle, WA 98195; ^dd^Laboratory for the Modeling of Biological and Socio-technical Systems, Network Science Institute, Northeastern University, Boston, MA 02115; ^ee^Operations Research and Industrial Engineering, The University of Texas at Austin, Austin, TX 78712; ^ff^Department of Infectious Disease Epidemiology, Faculty of Epidemiology and Population Health, London School of Hygiene & Tropical Medicine, London WC1E 7HT, United Kingdom; ^gg^Centre for Mathematical Modelling of Infectious Diseases, London School of Hygiene & Tropical Medicine, London WC1E 7HT, United Kingdom; ^hh^South African Department of Science and Innovation - National Research Foundation Centre of Excellence in Epidemiological Modelling and Analysis, Stellenbosch University, Stellenbosch, 7600 South Africa; ^ii^Texas Advanced Computing Center, The University of Texas at Austin, Austin, TX 78712; ^jj^National Institute of Standards and Technology, Gaithersburg, MD 20899; ^kk^School of Natural Resources and Environment, University of Florida, Gainesville, FL 32611; ^ll^The 28th Research Institute of China Technology Group Corporation, Nanjing, Jiangsu 210023, People’s Republic of China; ^mm^School of Agriculture and Environment, University of Western Australia, Perth, WA 6009, Australia; ^nn^Zeeman Institute for Systems Biology and Infectious Disease Epidemiology Research, School of Life Sciences and Mathematics Institute, University of Warwick, Coventry, CV4 7AL, United Kingdom; ^oo^Centers for Disease Control and Prevention COVID-19 Response, Atlanta, GA 30329; ^pp^Department of Biomedical Informatics, School of Medicine, University of Pittsburgh, PA 15260; ^qq^U.S. Geological Survey, Eastern Ecological Science Center, Laurel, MD 20708

**Keywords:** multi-model aggregation, decision theory, cognitive biases

## Abstract

Policymakers must make management decisions despite incomplete knowledge and conflicting model projections. Little guidance exists for the rapid, representative, and unbiased collection of policy-relevant scientific input from independent modeling teams. Integrating approaches from decision analysis, expert judgment, and model aggregation, we convened multiple modeling teams to evaluate COVID-19 reopening strategies for a mid-sized United States county early in the pandemic. Projections from seventeen distinct models were inconsistent in magnitude but highly consistent in ranking interventions. The 6-mo-ahead aggregate projections were well in line with observed outbreaks in mid-sized US counties. The aggregate results showed that up to half the population could be infected with full workplace reopening, while workplace restrictions reduced median cumulative infections by 82%. Rankings of interventions were consistent across public health objectives, but there was a strong trade-off between public health outcomes and duration of workplace closures, and no win-win intermediate reopening strategies were identified. Between-model variation was high; the aggregate results thus provide valuable risk quantification for decision making. This approach can be applied to the evaluation of management interventions in any setting where models are used to inform decision making. This case study demonstrated the utility of our approach and was one of several multimodel efforts that laid the groundwork for the COVID-19 Scenario Modeling Hub, which has provided multiple rounds of real-time scenario projections for situational awareness and decision making to the Centers for Disease Control and Prevention since December 2020.

## Significance

During infectious disease outbreaks, uncertainty hinders our ability to forecast dynamics and to make critical decisions about management. Motivated by the COVID-19 pandemic, leading agencies have initiated efforts to prepare for future outbreaks, for example, the US Centers for Disease Control and Prevention’s National Center for Forecasting and Outbreak Analytics and the WHO’s Hub for Pandemic and Epidemic Intelligence were recently inaugurated. Critically, such efforts need to inform policy as well as provide insight into expected disease dynamics. We present a validated case study from early in the pandemic, drawing on recommendations to minimize cognitive biases and incorporate decision theory, to illustrate how a policy-focused process could work for urgent, important, time-sensitive outbreak decision making in the face of uncertainty.

Uncertainty is pervasive during any infectious disease outbreak. There is limited scientific understanding about epidemiological processes; public health and economic goals may be varied, unclear, conflicting, or not stated at all, and the potential effects of possible interventions are uncertain given the novel circumstances. The risks associated with making a decision in the face of uncertainty are a central feature of the decision maker’s dilemma. As illustrated by recent outbreaks of Ebola and Zika viruses, and the COVID-19 pandemic, the complexity of an outbreak and a desire to help motivates quantitative modeling, but a profusion of models often produces conflicting forecasts, projections, and intervention recommendations (1–9).

**Fig. 1.**
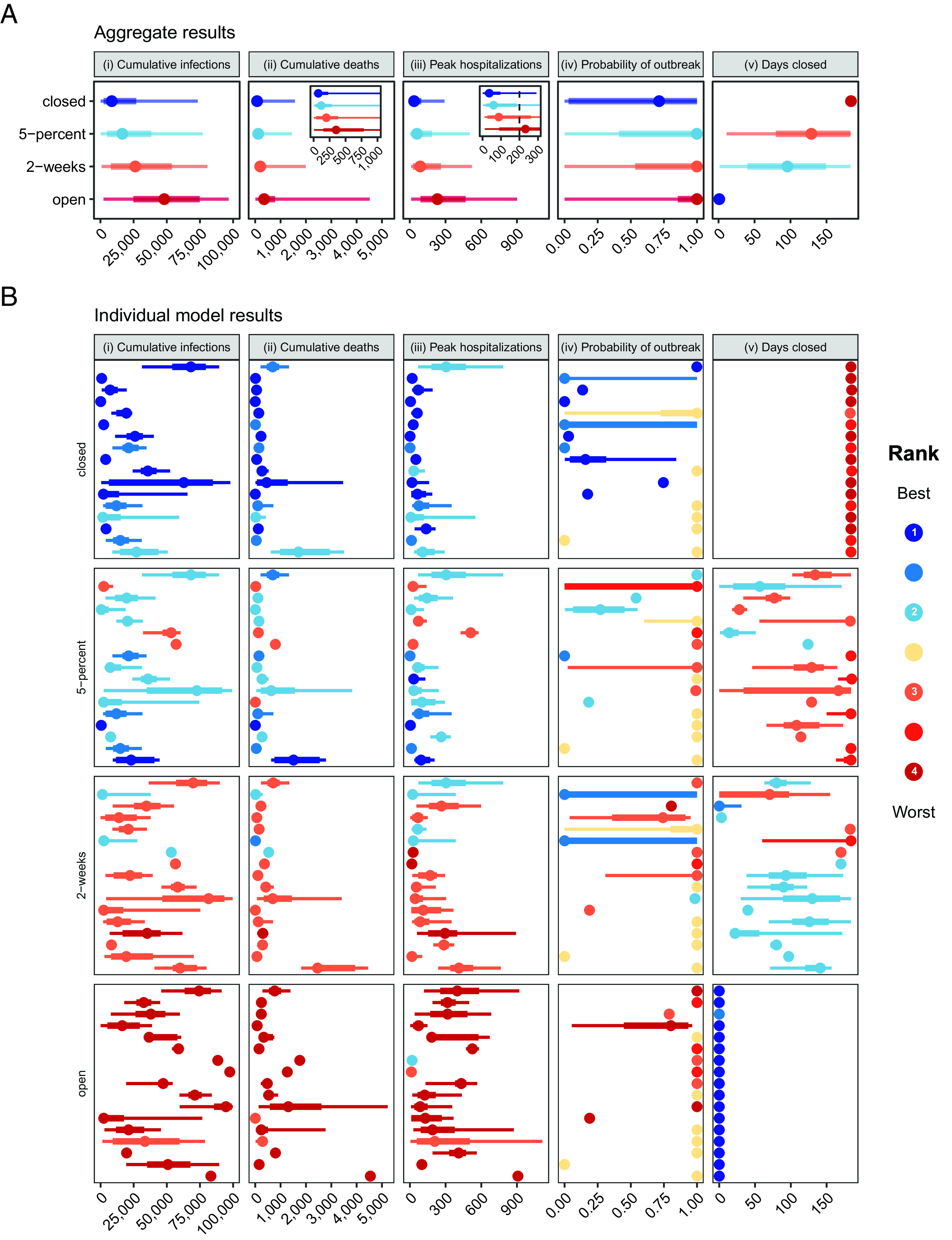
Aggregate and 17 individual model results for target objective and intervention scenario pairs. Medians, 50% prediction intervals (PIs, the range of values within which we project future outcomes will fall with 50% probability), and 90% PIs are indicated as points, thick lines, and thin lines, respectively. Colors denote ranking of each intervention for a single objective, where dark blue signifies the lowest value (best performance) and dark red signifies the highest value (worst performance). Ties in ranks are colored as intermediate values. Ties between ranks 1 and 2 and ranks 3 and 4 are shown as an intermediate blue and red, respectively; yellow indicates a tie in ranks across all interventions. The five panels show the results for: i) cumulative SARS-CoV-2 infections (rather than reported cases) between May 15 and November 15, 2020; ii) cumulative deaths due to COVID-19 over the same period, with an inset displaying the results for a smaller range of values, beginning with zero and containing the 50% prediction intervals; iii) the peak number of hospitalizations over the same period, with the inset showing a smaller range of values, and the hospital capacity of 200 beds with a vertical dotted line; iv) the probability of an outbreak of greater than 10 new cases per day after May 15; and v) the number of days that nonessential workplaces are closed between May 15 and November 15. The interventions include “closed”, workplace closure throughout the 6-mo period; “5-percent”, nonessential workplace reopening when cases decline below 5% of the peak caseload; “2-wk”, nonessential workplace reopening 2 wk after the peak; and “open”, immediate reopening of all workplaces on May 15. The setting is a generic US county of 100,000 people that had experienced 180 reported cases and 6 deaths as of May 15, 2020; all schools are assumed to be closed throughout the projection period. (*A*) Aggregate distributions for each objective and intervention scenario pair. The aggregate distributions were calculated as the unweighted average of the individual cumulative distribution functions across the 16 modeling groups. (*B*) Individual model results for each objective and intervention scenario pair. Results are presented here essentially as they were shown to modeling teams during the between-round discussion (except each projection was labeled with a letter that was only shared with the team that generated that projection); projections were intentionally presented anonymously to avoid groupthink and authority bias.

We integrated established methods from decision analysis (10), expert elicitation (11–14), and model aggregation (5, 15, 16) to harness the power of multiple models (2) (see *SI Appendix*, Fig. S1 for an overview of the full process). We convened multiple, independent modeling teams to evaluate nonessential workplace reopening strategies for a generic mid-sized US county of approximately 100,000 people that closed during a COVID-19 outbreak in April–May 2020. At the time, control of SARS-CoV-2 in such populations had received relatively little attention but was relevant to decisions faced by state and local officials. We solicited models to project the impact of four reopening interventions over a 6-mo period for five management objectives related to SARS-CoV-2 morbidity and mortality (see *Materials and Methods* for details). The four possible interventions mirrored the range of COVID-19 responses observed in April 2020, focusing on the conditions for nonessential workplace reopenings, with schools remaining closed (Fig. 1*A*). We requested that each modeling group provide a probability distribution of health outcomes and proxies of economic burden for each intervention, from which aggregate results were generated. After the first round of projections, aggregate results and individual results (anonymized to minimize cognitive bias) were shared with all groups and a structured discussion with an independent facilitator was held to clarify terminology, share insights, and discuss differences (*SI Appendix*, Fig. S1). The discussion was designed to reduce unwanted cognitive biases and linguistic uncertainty (e.g., about the interpretation of the problem setting), while characterizing and preserving genuine scientific uncertainty (e.g., about epidemiological processes or parameters, or intervention efficacy, given limited data) that is relevant to policy development and decision making (2). Following the discussion, the groups provided updated projections, from which the final results were generated (see Dataset S2 for anonymized output data).

## Aggregate Results Anticipate Outbreaks for Any Level of Reopening

Sixteen modeling groups participated in this study, contributing 17 distinct models with a variety of structures and assumptions (see *Materials and Methods* and *SI Appendix*, *Supplementary Discussion*, Figs. S18 and S19, and Tables S1 and S2 for more information on the individual models). Most of the models were compartmental or agent-based; the majority addressed age structure in some way. Models used a wide range of different methods to handle different sources and levels of uncertainty, but all participants were able to provide 100 quantiles for each objective-intervention combination (i.e., we requested the probability distribution for each outcome for each intervention). These independent probabilistic submissions were then aggregated to account for uncertainty within and between individual projections, using a linear opinion pool approach (17) and equal weighting (18). The aggregate projections showed a consistent ranking of intervention performance (*SI Appendix*, Figs. S2 and S9 and Movie S1) across the four public health outcomes (cumulative infections, cumulative deaths, peak hospitalization, and probability of an outbreak), and a strong trade-off between public health and economic outcomes (number of days with nonessential workplaces closed over the 6-mo period, Fig. 1*A*). For all public health-related outcomes, the best intervention was to keep nonessential workplaces closed for the duration of the period investigated. Reopening when cases dropped to 5% of the peak and reopening 2 wk after the peak were ranked second and third, respectively. Opening fully and immediately led to the greatest public health burden. Keeping restrictive measures in place for 6 mo reduced median cumulative infections by 82%, from 48,100 (48.1% of the county population) in the open intervention to 8,527 in the closed intervention; delaying reopening to 5% of the peak or 2 wk after the peak reduced the cumulative infections by 66% and 46%, respectively, relative to the open intervention. The reduction in cumulative deaths followed a similar pattern (Fig. 1*A*). Peak hospitalizations ranked the interventions in the same order as cumulative cases and deaths, but the largest decrease was achieved when going from the open intervention to the 2-wk intervention. Any reopening of nonessential businesses triggered a second outbreak; even when workplace restrictions were maintained for 6 mo, the probability of an outbreak was high (aggregate median, 71%). Our study suggests nonessential workplace closures alone would have been insufficient to interrupt transmission of COVID-19 at a county level. This corresponds to global observations–only a few countries (e.g., New Zealand, Taiwan) succeeded in preempting or extinguishing COVID-19 outbreaks in the prevariant and prevaccine era, using strong compliance to social distancing and travel restrictions.

Our results explicitly demonstrate trade-offs between economic and public health outcomes; how much economic activity might the decision maker be willing to forgo to gain public health improvements of a given magnitude? The number of days of nonessential workplace closure is a coarse and incomplete measure of short-term economic impact, but it highlights an important trade-off (see *SI Appendix*, *Supplementary Text: Tradeoff between public health and economic objectives* for a discussion of further considerations for a comprehensive epidemiological-economic analysis). The ranking of interventions in relation to projected days closed was reversed in comparison to the ranking for each public health outcome (Fig. 1*A*): Under the closed intervention, nonessential workplaces were closed for 184 d (May 15 to November 15); under the 5-percent and 2-wk interventions, nonessential workplaces were closed for a median of 129 and 96 d (a reduction of 29% and 48%), respectively. We had hypothesized that the 5-percent intervention might be an attractive alternative relative to remaining closed, permitting a reduction in days closed with little or no difference in public health outcomes. However, no win-win intermediate reopening strategies were identified (Fig. 1*A*).

## Demonstrated Benefits of the Structured Multiple Models for Outbreak Decision Support (MMODS) Process

**Fig. 2.**
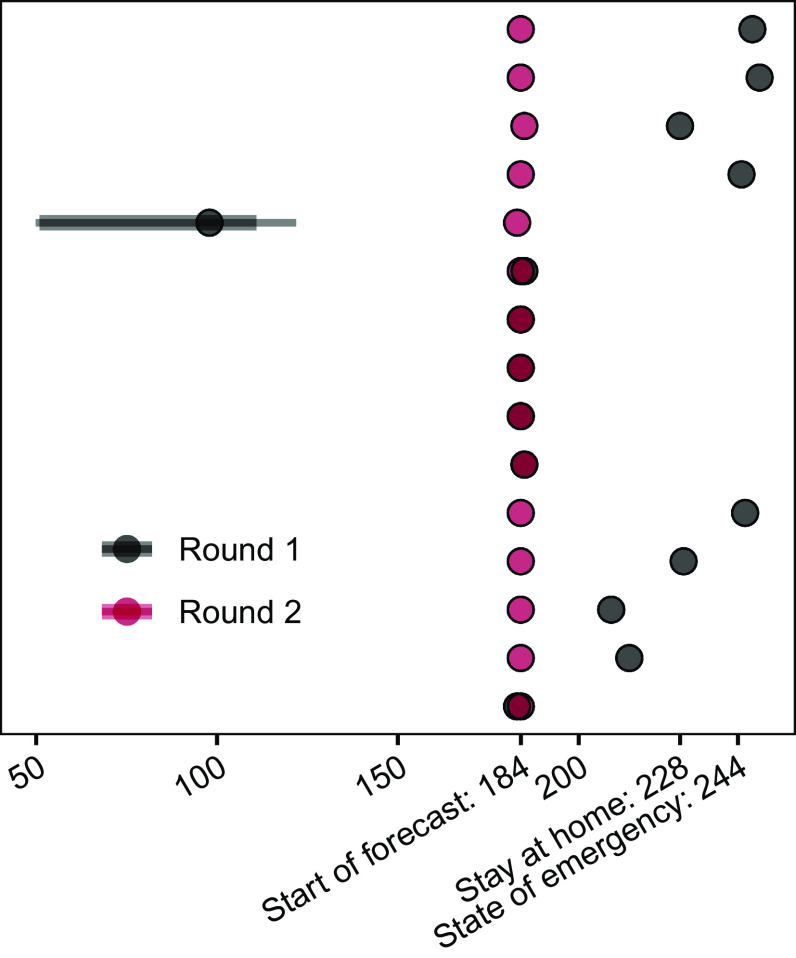
Resolution of linguistic uncertainty in the discussion following round 1 of modeling about the number of days nonessential workplaces are closed. Direct comparison of round 1 and 2 results for days closed for the fully closed intervention. In round 1, groups used a variety of start dates (start of the forecast, first day of stay at home orders, or state of emergency declarations) and one group implemented a weighting for essential and nonessential business closures and associated compliance issues explicitly.

The MMODS process is focused on decision outcomes, providing aggregate, rapid results to a decision maker. The process allows for the interim use of a first round of results if a rapid decision is required, but a second round of results, updated after discussion with all teams, provides the most benefit. Two rounds of modeling, with an intervening discussion (to allow for clarifications and sharing of insights between modeling teams and facilitators), removed multiple confusions about interventions and objectives in our study (*SI Appendix*, *Supplementary Discussion: Resolution of Linguistic Uncertainty*). The discussion also motivated the modification of one reopening intervention (*SI Appendix*, Fig. S1 loop A; *Materials and Methods*). The discussion highlighted additional sources of available data, shared critical insights with all groups, and encouraged a broader expression of scientific uncertainty, all while maintaining anonymity of results to avoid sources of cognitive bias (11–14) such as groupthink (i.e., conforming to the group without sufficient critical evaluation) and dominance effects (i.e., attributing more attention to the opinions of figures of authority). As linguistic uncertainty was decreased at the same time as modeling groups were encouraged to more fully express remaining scientific uncertainty, there was no expectation of directionality in the relative magnitude of uncertainty expressed by models in the two rounds of projections. However, projections of days closed in the two extreme (open and closed) interventions were highly variable in round 1, due to different misinterpretations of the metric, but were entirely consistent across modeling groups in round 2 (Fig. 2), demonstrating the importance of removing or reducing linguistic uncertainty that can severely confound results.

## Individual Models Are Consistent in Ranking of Interventions, but Projections Are Variable in Magnitude and Uncertainty

The rankings of interventions from the individual models are generally consistent with each other and with the aggregate results for a given objective (Fig. 1*B* and Movie S1). The consistent ranking was unexpected given the uncertainty about intervention efficacy at the time [though also seen in Ebola (5)] and stands in distinct contrast to the considerable variation in the magnitude and uncertainty of projections displayed by the individual models (Fig. 1*B*). For example, cumulative infections for the full reopening strategy ranged widely, despite all models ranking the intervention as worst. Reliance on a single model, rather than an ensemble, is inherently less reliable for providing insights into the magnitude of differences between interventions, even if the ranking of interventions is relatively robust (*SI Appendix*, Fig. S2). Where models disagree on ranking (e.g., for probability of a second outbreak), the aggregate generates a clear ranking that encapsulates the uncertainty. Differences in model structure, parameterization, and assumptions that the groups were asked to provide did not immediately explain differences in projections (*Materials and Methods* and *SI Appendix*, Tables S1 and S2 and Figs. S18 and S19); such an evaluation would be impossible with individual experts. Disagreements between models, or between models and the aggregate, were examined retrospectively, and arise for a range of reasons: Genuine scientific disagreement about processes to include in the model given the massive uncertainty about SARS-CoV-2 at the time; stochasticity (especially in the case of very close or “tied” results); differences in calibration approaches; residual linguistic uncertainty; and inclusion of assumptions groups would choose to revise (with benefit of hindsight).

## Aggregate Results Provide an Integrated Expression of Uncertainty

It is challenging for any individual model alone to fully account for uncertainty. The aggregate results provided a more comprehensive expression of uncertainty in projected outcomes by integrating over projected outcomes from individual teams with varying assumptions about disease dynamics, population behavior, public health surveillance, and the effectiveness of interventions. Deploying multiple models in parallel also speeds up the process of exploring relevant uncertainty. Between-model variation was substantial and similar to or greater than variation within any single model. Individual models tended to capture less than 50% of the uncertainty of the aggregate (as measured by the relative interquartile range (IQR): see *Materials and Methods* and *SI Appendix*, *Supplementary Discussion* and Fig. S10). Thus, individual models were generally more confident than the ensemble, echoing findings from studies of expert judgment that individual experts tend to be overconfident (19). The ensemble provided valuable risk quantification for decision making (20) since all models provided projections as probability distributions. For example, for a county with 200 hospital beds, although median peak hospitalization is comparable for the remain-closed and 2-wk interventions, the 2-wk intervention was three times as likely to exceed capacity as the remain-closed intervention (34% vs. 11% chance of exceedance based on the aggregate results, Fig. 1*A*). These risk estimates allow the administrator to gauge how much to prepare for exceedance.

## Model Projections Are Comparable with Real-World Data

**Fig. 3.**
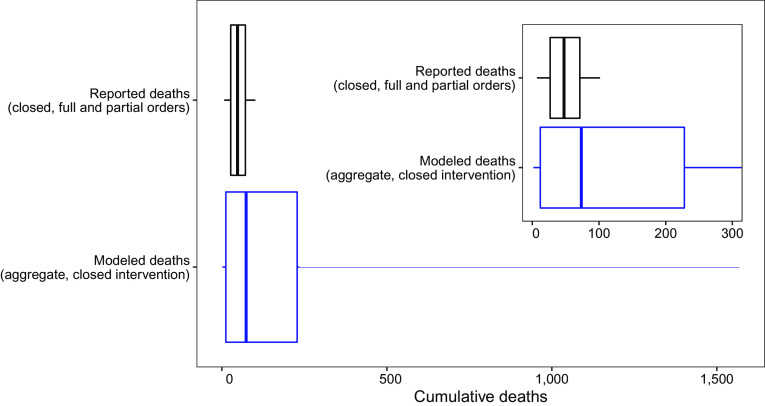
Comparison of aggregate reported county death data to modeled deaths for the closed intervention. Boxplot of cumulative reported deaths from 84 US counties with full or partial stay at home orders in place from May 15 to November 15, 2020 (median deaths: 48; 50% IQR: 27, 71) and model results for cumulative deaths from May 15 to November 15, 2020 under the closed intervention (median deaths: 73; 50% IQR: 12, 228). Vertical line shows median value, box shows IQR (25th to 75th quantiles), and whiskers extend to the 5th and 95th quantiles. *Inset* shows overlap of box area for the plots.

We identified 84 mid-sized (90,000 to 110,000 people) US counties that approximated the profile of the setting presented to the modeling groups and that implemented and followed a closed intervention (e.g., a stay-at-home order) through November 15, 2020 (21) (*SI Appendix*, *Supplementary Discussion: Comparison of county death and case data with aggregate model results*). The distribution of reported deaths due to COVID-19 in the closed counties (median deaths during the projection period 48; 50% IQR, 27 to 71; Fig. 3, black) is comparable to the aggregate projection of total deaths (median deaths 73; 50% IQR, 12 to 228; Fig. 3, blue). The prediction intervals for deaths (and cases) were wider than the observed distributions, which is expected as the observations represent a subset of the possible paths that the outbreak might have taken (*SI Appendix*, Figs. S2, S3, S4, S16, and S17 and *Supplementary Discussion: Comparison of county death and case data with aggregate model results*).

## Discussion

The abundance of uncertainty that accompanies pathogen emergence presents a difficult challenge for public health decision making (1, 2, 22). However, we show that aggregate results from a multistage, multimodel process ranked interventions consistently for the objectives we considered. Unfortunately, while more stringent reopening rules generally performed better, strategies designed only around one-time reopening guidelines were inadequate to control the COVID-19 epidemic at the county level, as reflected in the resurgence of COVID-19 over the summer of 2020 in the United States. Our Centers for Disease Control and Prevention (CDC) collaborators reported that this “unique collaboration … provided strong, timely evidence that control of the COVID-19 pandemic would require a balance of selected closure policies with other mitigation strategies to limit health impacts” (3).

The descriptions of the objectives and interventions for this elicitation were motivated by public discussions and guidance issued by US federal and state governments in April 2020. However, concepts presented in colloquial language can be difficult to precisely define mathematically. All groups found that the initial wording in the guidance provided was difficult to interpret and model, suggesting that it could invite considerable discretion in implementation. Our process identified potential ambiguities, and we provided a clear definition of such terms for the second round of projections (see *Materials and Methods* for definitions, and *SI Appendix*, *Supplementary Discussion: Resolution of Linguistic Uncertainty* for discussion). Establishing clear lines of communication and open, structured collaboration between decision makers and modelers (*SI Appendix*, Fig. S1, loop A), would reduce confusion and permit consistent evaluation of management interventions.

The MMODS approach provides valuable lessons for the process of eliciting projections from multiple modeling teams to inform decision-making. Running the models at least twice, with an intervening discussion, is essential. In our between-round discussion, we removed different interpretations of terminology that would have confounded individual model comparisons and the aggregate results. For example, the trade-off between days closed and public health outcomes would have been obscured by linguistic uncertainty surrounding “closure” (Fig. 2). Initially, it might seem that adding a second step would delay decision making; in an emergency, results from round 1 could be used to inform an interim decision. However, clarification often happens on an ad hoc basis anyway, as it is fundamentally difficult to anticipate everything that groups might interpret differently a priori, and uncertainties often arise as models are developed and implemented. Our process saves time by avoiding multiple, unplanned reassessments and would be particularly valuable in situations where the same models are used to make repeated decisions. Our case study generated first-round results about a month after the start of the multimodel project and produced final results about a month later (*Materials and Methods*). This pace can be accelerated with experience; the COVID-19 Scenario Modeling Hub conducted 13 rounds in just over a year, with repeat emergency Omicron results generated just over 2 wk apart (23). Resolution of linguistic uncertainty via facilitated group discussions would likely also improve forecasts outside the decision-making setting.

Multiple model approaches such as MMODS come with challenges. Most of these challenges could be addressed via planning and preparedness efforts to build a cohesive community of epidemiological and decision-making experts in anticipation of need (24–27). Such coordinated efforts could then provide timely, informed support to decision makers at critical junctures, to enable effective decision making in the face of uncertainty. This approach can then be applied to a wide range of settings or to other critical decisions, at any level of governance from the local to the international. At the local level, nearly every medical system in the world faced unprecedented hospital demand during the pandemic, and many struggled to cope; our hospital risk analysis has broad application to planning for local public health resources everywhere, including in low- and middle-income countries (LMICs). At the national level, decisions for which the MMODS approach could be used include the following: when to reimpose or relax interventions in a sequence; context-dependent state- and country-level interventions (including in LMICs where constraints such as resource availability may differ markedly on a case-by-case basis, e.g., ref. 28); where best to trial and allocate improved vaccines and drugs; and how to prioritize testing to enable economic reopening earlier in an outbreak. At the national and international levels, the MMODS approach can also be used to develop guidelines that optimally deploy non-pharmaceutical interventions (NPIs) and how to optimize the roll-out of vaccines and other interventions. In fact, the MMODS case study presented here was one of several multimodel efforts that laid the groundwork for the US COVID-19 Scenario Modeling Hub (23). The Hub has provided multiple rounds of real-time scenario projections, addressing uncertainty about vaccine coverage, NPI compliance, variant characteristics, and waning immunity, to the US CDC since December 2020 (e.g., refs. 3 and 29–32).

**Fig. 4.**
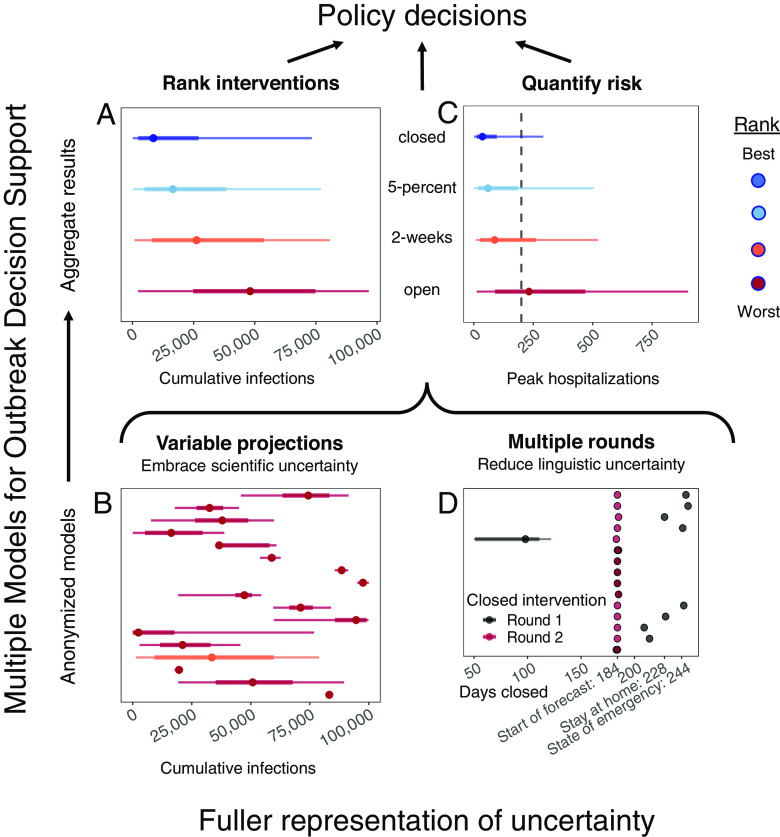
Overview of the MMODS approach: Embracing uncertainty to inform COVID-19 nonessential workplace reopening decisions using multiple models for outbreak decision support. Aggregate results ranked interventions consistently for specific objectives (e.g., minimizing cumulative infections) (*A*) even though the model-specific magnitude of projections varied greatly (*B*). Ensemble aggregate distributions quantify risk for decision makers and enable them to inform decisions in the context of resource limitations, such as the availability of hospital beds denoted by the vertical dotted line (*C*). An intermediate discussion between two rounds of independent model projections resolved inconsistencies in interpretation of intervention implementation as well as objective specification (*D*).

Nevertheless, significant challenges remain to effectively using our approach in formal decision making. Our open study used multiple models, instead of multiple individual experts, in a structured expert elicitation process deliberately designed to reduce cognitive biases that commonly occur in group decision making, and addressed real-time disease mitigation decisions (see the MMODS overview in Fig. 4). However, our effort focused nearly entirely on public health benefits, with little reference to other essential features of a full cost-benefit analysis addressing economic costs and possible trade-offs between public health and economic objectives (*SI Appendix*, *Supplementary Discussion*). Additionally, many decision makers have other (e.g., social) objectives, which may be challenging to elicit and quantify, especially in time-sensitive situations. How do both explicit and unspoken objectives affect the design of scenarios and the resultant outputs? While we have drawn from the expert judgment literature (12, 33) to design this process, more work is needed to streamline the approach (2). For example, does an open call for participation or a curated set of established models produce better results? How many models are needed to gain sufficient expression of between-model uncertainty and produce stable and robust results? In individual expert judgment approaches, between 5 and 20 experts are recommended (34); however, it is not clear if the same applies for models. Should we consider all model outputs equally? Equal weighting is typically robust, and simple rules may be favored (18). However, if the MMODS decision process were used to guide repeated decisions (*SI Appendix*, Fig. S1, loop C), there is the opportunity to update the weights on individual models in the ensemble as we learn about their performance and for modeling teams to learn from surveillance and auxiliary data and improve their models. Conscious a priori examination of critical uncertainties, using value of information analyses, could provide the foundation for an adaptive management framework to improve understanding and management outcomes over time (2, 35–37). What is the best way to aggregate model results for different purposes (17)? Is our result that fewer models were needed to provide robust ranking of interventions than were needed to generate reliable projections of the magnitude of disease burden (cases, hospitalizations, deaths) general? Answering these questions would help us increase efficiency in future crises.

Our MMODS study demonstrates clear benefits of a real-time, decision-focused, collaborative modeling process that carefully handles multiple sources of uncertainty and mitigates cognitive biases common in group judgments via purposeful, structured communication. The MMODS approach can also be used for critical management decisions for endemic diseases, in data-poor settings, for elimination and eradication planning, as well as in any nonepidemiological setting where models are used to inform decision making (e.g., ref. 38). In our case study, multiple contributors from federal agencies and academic institutions collaborated to address a common problem. Our approach could be a valuable step in a revised long-term strategy for responding to recurrent outbreaks and pandemics, with the potential to save lives and reduce suffering.

## Materials and Methods

We solicited participation from modeling groups via the Models of Infectious Disease Agent Study (MIDAS) network and via existing COVID-19 modeling collaborations with the US CDC facilitated by the MIDAS Coordination Center at the end of May 2020. Information about the collaboration opportunity was communicated via conference calls and listservs. This activity was reviewed by CDC and was conducted consistent with applicable federal law and CDC policy (45 C.F.R. part 46, 21 C.F.R. part 56; 42 U.S.C. §241(d); 5 U.S.C. §552a; 44 U.S.C. §3501 et seq).

We presented information for a generic, mid-sized county of approximately 100,000 people with age structure representative of the US population, that preemptively initiated, and adhered to, stringent social distancing guidelines (i.e., full stay-at-home orders with workplace and school closures) until May 15, 2020 (so that the 6-mo prediction period ran from May 15-November 15, 2020). We provided the modeling groups with baseline epidemiological and intervention information for the county (see Dataset S1 containing provided data); some groups incorporated additional data (*SI Appendix*, Table S1).

Full information for the elicitation, including the setting, epidemiological data, and intervention descriptors was posted at a dedicated website at https://midasnetwork.us/mmods/ (including daily reported cases, deaths, mobility, and testing data, State of Emergency and stay-at-home orders, and age structure). The initial conditions for the projections included cumulative cases and deaths within the county on a daily basis from January 22, 2020 to May 15, 2020. As of May 15, the county had recorded 180 confirmed cases and six deaths due to COVID-19. Groups were also permitted to incorporate additional datasets, as they saw fit (e.g., national data on hospital, Intensive Care Unit and ventilator availability, household size data, and work, school, community, and home mixing data). We asked the modeling groups to assume that travel restrictions remained in place throughout (so that there was no international importation of cases and domestic importations were limited) and that there was no local contact tracing or isolation of infected individuals. We did not specify guidelines regarding mask use but specified that schools would remain closed through November 15, 2020 (just prior to the start of peak flu season). First-round results were due on June 15, 2020, and the group discussion of preliminary results took place on June 24, 2020. Second-round model results were due July 12, 2020, and preliminary analyses of second-round results were reported to the modeling groups and others on July 17, 2020.

The five metrics corresponding to management objectives were 1) cumulative number of infected individuals (May 15 to November 15); 2) cumulative number of COVID-related deaths over the same period; 3) peak hospitalizations during the period May 15 to November 15; 4) probability of a new local outbreak after May 15 (more than 10 new reported cases per day); and 5) total number of days workplaces closed. The four interventions focused on strategies for reopening nonessential workplaces, while assuming that all involved schools remaining closed 1) continue with current nonessential workplace closures at least through November 15 (“closed intervention”), 2) open nonessential workplaces when the number of new daily reported cases is at 5% of peak (“5-percent intervention”), 3) open nonessential workplaces 2 wk after peak (“2-wk intervention”), and 4) immediately relax all current restrictions on nonessential workplaces on May 15 (“open intervention”).

All objective-intervention combinations were assessed for feasibility in an in-house model prior to developing the elicitation. However, intervention 2, which was set at 1% in round 1, was identified as too restrictive (i.e., the condition was never met) by several models during the discussion and was therefore changed accordingly (i.e., modeling groups provided feedback on interventions as anticipated in *SI Appendix*, Fig. S1 loop A). In a single-round elicitation, such a situation would have effectively reduced the number of interventions examined overall, as the 1% trigger was essentially congruent with the fully closed intervention.

For the 5-percent and 2-wk interventions (interventions 2 and 3), following the resolution of linguistic uncertainty in the discussion (*SI Appendix*, *Supplementary Text: Resolution of linguistic uncertainty*), we asked all groups to use the same metric and method for calculating the peak, acknowledging that this is only one of several metrics and methods that could be used to determine the peak. We chose a definition that could be implemented by a decision maker (as opposed to an omniscient approach). For both the 2-wk and 5-percent interventions, all teams used the 7-d trailing moving average of the number of new daily reported cases (as opposed to all infections, which may or may not result in reported cases); the moving average smooths out noise due to reporting and low population size. Peak is then defined as the maximum 7-d moving average of daily reported cases. The trigger to open for the 2-wk intervention is the first day for which the 7-d trailing moving average has been lower than the maximum for at least 14 d and has shown a day-to-day decline in smoothed case data for at least 10 of the last 14 d (or there have been 7 d without any new cases). The trigger to open for the 5-percent intervention is the first day for which the 7-d trailing moving average of the number of new daily reported cases drops below 5% of the peak after May 15th. Note that the peak that triggers a 2-wk intervention may not be the same peak that triggers a 5-percent intervention (e.g., if there is a second peak that is larger than the first one).

Each group completed a thorough model description checklist (*SI Appendix*, Table S1) for each round, to document a wide range of information on model structure and parameterization, the efficacy of interventions, additional setting information, assumptions and the associated uncertainty, as well as other sources of stochasticity (*SI Appendix*, Tables S1 and S2 and Figs. S18–S20). Of the 17 models contributed by 16 scientific research groups, 10 were compartmental, 5 individual-based, 3 spatially explicit, 1 neural network, and 1 fractional order model. Eleven models included age-structure explicitly for some model components. Modeling teams individually handled uncertainty in a variety of ways, using different methods and addressing different components (e.g., expert judgment for choice of model structure and parameters, likelihood-based methods for parameter estimation, or simulation-based approaches for exploring ranges of potential parameter values). As part of the submission checklist, model groups were asked to provide an estimate of the number of full-time equivalent (FTE) hours allocated to their modeling effort, so we could explicitly specify the human resources required to undertake such a multimodel effort where there are 17 models. Modeling groups allocated an estimated 64 median FTE hours (Q1: 40 FTE hours, Q3: 100 FTE hours, Max: 1,000 FTE hours). No groups dropped out between rounds. One group did not submit a model for round 1 but participated in the discussion and submitted to round 2.

We requested 100 quantiles for each model–objective–intervention combination such that tail probabilities for the 2nd and 98th quantiles were relatively stable [i.e., we requested the probability distribution for each outcome for each intervention, via the cumulative distribution function (CDF) in 100 quantiles]. Requesting quantiles (rather than, for example, epidemiological curves) enables all types of different models to participate and allows a better expression of uncertainty for decision making. Collecting 100 quantiles allows the tail probabilities to be estimated. We deliberately did not request information on the correlation structure between interventions within a model, as not all models were equipped to provide results for the same initial conditions or seed values.

Submissions were received by the MIDAS Coordination Center through the MMODS website, verified for format compliance, transformed into a consistent format for analysis, and deposited in an internal project GitHub repository. Deadlines and submission times were documented, enabling us to explicitly specify the time frame within which the two-round process can occur. Anonymized model submissions, as well as aggregation, analysis, and figure generation code can be found in the public repository: https://github.com/MMODS-org/Elicitation-1 (39).

Aggregate results were produced by taking a weighted average of the individual CDFs (*SI Appendix*, Fig. S8); this provides critical information about the mean as well as higher-order moments. Each group received equal weight in the aggregate results. For research groups submitting more than one model, the group weight was divided equally among their models (one group submitted two models). Based on the detailed checklist information, we can explicitly document the differences between models and the CDFs reflect the full degree of uncertainty considered. This model-based approach presents two advantages over human experts, who generally provide 3 to 5 quantiles at most and generally do not document explicit differences in their thought processes that might generate different rankings (37). While it is impossible to do a full analysis of every difference between all the models, we explored multiple potential correlates of model result rankings and magnitudes. Ancillary information was examined to assess whether model assumptions (e.g., model structure, assumptions about importations, etc.) predicted ranks or magnitudes of projections, or various other aspects of epidemic dynamics (e.g., projected number of people who are susceptible on November 15, 2020): nothing obvious emerged (*SI Appendix*, Figs. S18–S20 and Table S1). There also was no obvious uncertainty that would reverse the choice of optimal intervention (e.g., a factor whose inclusion or exclusion leads to different rankings); had such a factor arisen, this would be a top priority for research to improve decision-making outcomes. We also document where differences in magnitude between two strategies are not large; this is only the case for individual models, but not in the aggregate. In such cases, the decision maker may have flexibility of choice and may choose to weigh other considerations (such as costs) that have not been explicitly included in the models. Magnitude was not our primary interest but is important in determining whether the overall benefits of an intervention are sufficient to outweigh the overall costs.

Participating modeling groups refocused their efforts on COVID-19 during the pandemic and contributed considerable, mostly unfunded, time and effort (*SI Appendix*, Table S1) to participate in this project on a voluntary basis. Not all models were initially structured to address the questions or interventions we considered, and the methods for handling uncertainty were new for some of the groups. Additionally, we found some trade-offs were necessary. A number of the models (e.g., those with substantial simulation time or requiring extensive changes to implement new interventions) could not assess the impact of many distinct interventions so we limited our study to four workplace-related interventions to encourage participation. However, this need not constrain the number of interventions for critical decisions and situations; it is possible to augment computational resources, or different subsets of the contributing models could assess different subsets of interventions. The success of future collaborative efforts will depend on the sustained availability of financial and logistical support, for both the coordination of collaborations, and for the individual modeling groups (26).

## Supplementary Material

Appendix 01 (PDF)

Dataset S01 (XLSX)

Dataset S02 (XLSX)

Movie S1. **MMODS_Elicitation1_InterventionRankResults.mp4** Model-specific intervention rank results evaluated for each objective in round 2 of the MMODS process. Results are displayed in video format by quantile (1 through 100). Colors indicating ranks and rank-ties are as specified for the median rank result figures in main text and range from single best intervention (dark blue) to single worst intervention (dark red).

## Data Availability

Anonymized data are available in the Dataset S2. Code is available on GitHub: https://github.com/mmods-org/elicitation-1 (39). USGS Disclaimer: Any use of trade, firm, or product names is for descriptive purposes only and does not imply endorsement by the US Government. CDC Disclaimer: The findings and conclusions of this report are those of the author(s) and do not necessarily represent the official position of the Centers for Disease Control and Prevention. NIST Disclaimer: These opinions, recommendations, findings, and conclusions do not necessarily reflect the views or policies of the National Institute of Standards and Technology or the United States Government. Food and Drug Administration Disclaimer: This article reflects the views of the group of authors and should not be construed to represent the FDA’s views or policies.
