# Supplementary material for: Multiple models for outbreak decision support in the face of uncertainty
Source: Proc Natl Acad Sci U S A. 2023 Apr 25;120(18):e2207537120. doi: 10.1073/pnas.2207537120 (PMC10160947; doi:10.1073/pnas.2207537120)
Supplement: Supplementary file 1 — Appendix 01 (PDF) [file pnas.2207537120.sapp.pdf]

Supplementary Materials for

COVID-19 reopening strategies at the county level in the face of uncertainty: Multiple Models for Outbreak Decision Support

Katriona Shea[1*†], Rebecca K. Borchering[1†], William J.M. Probert[2†], Emily Howerton[1†], Tiffany L. Bogich[1†], Shou-Li Li[3†], Willem G. van Panhuis[4†], Cecile Viboud[5†], Ricardo Aguás[2], Artur Belov[6], Sanjana H. Bhargava[7], Sean Cavany[8], Joshua C. Chang[9,10], Cynthia Chen[11], Jinghui Chen[12], Shi Chen[13,14], YangQuan Chen[15], Lauren M. Childs[16], Carson C. Chow[17], Isabel Crooker[18], Sara Y. Del Valle[18], Guido España[8], Geoffrey Fairchild[18], Richard C. Gerkin[19], Timothy C. Germann[18], Quanquan Gu[12], Xiangyang Guan[11], Lihong Guo[20], Gregory R. Hart[21], Thomas J. Hladish[7,22], Nathaniel Hupert[23], Daniel Janies[24], Cliff C. Kerr[21], Daniel J. Klein[21], Eili Klein[25, 26], Gary Lin[25], Carrie Manore[18], Lauren Ancel Meyers[27], John Mittler[28], Kunpeng Mu[29], Rafael C. Núñez[21], Rachel Oidtman[8], Remy Pasco[30], Ana Pastore y Piontti[29], Rajib Paul[13], Carl A. B. Pearson[31], Dianela R. Perdomo[7], T Alex Perkins[8], Kelly Pierce[32], Alexander N. Pillai[7], Rosalyn Cherie Rael[18], Katherine Rosenfeld[21], Chrysm Watson Ross[18], Julie A. Spencer[18], Arlin B. Stoltzfus[33], Kok Ben Toh[34], Shashaank Vattikuti[17], Alessandro Vespignani[29], Lingxiao Wang[12], Lisa White[2], Pan Xu[12], Yupeng Yang[26], Osman N. Yogurtcu[6], Weitong Zhang[12], Yanting Zhao[35], Difan Zou[12], Matthew Ferrari[1], David Pannell[36], Michael Tildesley[37], Jack Seifarth[1], Elyse Johnson[1], Matthew Biggerstaff[38], Michael Johansson[38], Rachel B. Slayton[38], John Levander[39], Jeff Stazer[39], Jessica Kerr[39], **Michael C. Runge[40†]**

*Correspondence to: k-shea@psu.edu
†Members of MMODS core team.

Supplementary Discussion

MMODS Motivation

To support sound, evidence-based decision making, we believe it is critical to develop an efficient framework for collaborative modeling and for synthesizing results and recommendations from ensemble modeling efforts (1, 2).

As with well-designed expert elicitations, using multiple models produces a more complete description of uncertainty and provides more robust projections to decision makers. Our approach can be applied to any decision that involves multiple models, and is designed to encourage full expression of scientific uncertainty while reducing extraneous uncertainty and minimizing biases common in elicitations from groups of experts. Many such decisions are being made worldwide in the context of COVID-19, for example.

Our approach is designed to reduce unwanted cognitive biases and linguistic uncertainty (e.g., about the interpretation of the problem setting), while characterizing and preserving genuine

scientific uncertainty (e.g., about epidemiological processes or parameters, or intervention efficacy, given limited data) that is relevant to policy development and decision making. In this framework, insights can be shared across modeling groups to inform the collective projections, while retaining the perspective of individual groups.

Sources of cognitive bias are many (3–6), but three main biases our process guards against include: dominance or authority effects (where there is a tendency to agree with field "leaders"); starting-point bias or anchoring (focusing on suggestions raised early in the process to the detriment of other ideas); and groupthink (when a desire for cohesiveness causes collaborators to minimize conflict and reach consensus without sufficient critical evaluation).

The process involves multiple steps (Fig. S1), including two rounds of modeling with an intervening structured discussion to eliminate unwanted biases and uncertainty (including semantic or linguistic uncertainty), increase consistency in modeling of interventions, share critical insights, and generate a comprehensive picture of relevant uncertainty. The projections from the second round of modeling are then used to generate aggregate results under different interventions that encapsulate scientific uncertainty about epidemiological processes and management interventions (7). We stress that this process is designed primarily to inform decision making, rather than to provide quantitative projections of epidemic trajectory (as in ongoing forecasting challenges (8)), though such results are also obtained. The multi-model, multi-step process is expected to generate better calibrated projections than individual models. That is, the aggregate distributional forecast will be more consistent with future observations than individual forecasts. More importantly, this process is also expected to produce more robust insights about the ranking of intervention options that improve management outcomes. In short, we use multiple models to generate better expressions of uncertainty while guarding against cognitive biases to provide decision support. The COVID-19 pandemic offers a unique opportunity to apply this structured framework.

We also stress that our approach encourages an integration of science and policy-making – efforts that are often separated to the detriment of public health, economic, and environmental outcomes when semantic uncertainties cannot be clarified and may thus interfere with success. Modelers intend their forecasts to 'inform management decisions,' yet the common separation of model outputs from the decision context increases the chance of misunderstandings and errors. Continued efforts to foster collaboration and streamline communication between modelers and decision makers, as well as to shift the focus from solely providing projections to evaluating proposed interventions, are essential steps towards effectively leveraging modeling efforts to inform decisions.

Resolution of linguistic uncertainty in structured discussion between rounds 1 and 2

The group discussion between modeling rounds identified numerous sources of linguistic uncertainty arising from different interpretations of the objectives and the nature of interventions from the problem setting. For example, the wording on reopening '2 weeks after peak' engendered considerable confusion in the first round of modeling. How is a peak defined? Is it in reported deaths or cases? Is it measured on a daily or a moving-average basis? Likewise, how

should a model determine whether 2 weeks have passed since the peak? A continuous monotonic decline was never seen; should a moving average be used? And, if so, for 7 or 14 days?

As well as allowing a common definition of "peak" and other terms, other sources of unanticipated uncertainty were resolved. For example, one modeling group asked for clarification on the definition of 'death.' There was a thorough discussion of the options that different groups had considered or used (reported only; reported plus probable; reported, probable and co-morbidities; or, also indirect deaths, such as those from unrelated causes in patients choosing not to go to the ER during a pandemic). We agreed as a group to use all deaths due to COVID-19 disease-induced mortality, regardless of reporting. This way of counting deaths is based on infection status, not testing status, and can include comorbidities but not indirect deaths, as we are only focusing on people who have been infected with SARS-CoV-2 and died from their infection.

The first round also provided some important checks and balances on the consistency of objective and intervention interpretations across groups, i.e., were the same definitions of workplace closures used? In the first round, some groups used the May 15 to November 15 timeframe, others based start dates on declarations of a State of Emergency or stay-at-home orders, and one group implemented a weighting for essential and non-essential business closures and associated compliance issues explicitly (Fig. S11). Including a metric that should be consistent across models allowed us to check for and remove linguistic uncertainty in round 2 submissions that would have limited our ability to compare the rankings of interventions between models and objectives. Clear guidelines developed during and after the group meeting removed this uncertainty from round 2 projections, improving the comparability of intervention rankings across models. Even so, there was still considerable variation across modeling groups in how these openings were triggered, in part because the triggering events were sensitive to how daily variation in the projections was handled (Fig. S12B). The MMODS Process is deliberate in explicitly planning for and appropriately managing this process, so that all groups are equally informed and use the same interpretations. Formally building the discussion phase into the modeling and decision-making process manages decision-maker expectations. Modeling teams also commented that they found the well-defined structure in Fig. S1 to be valuable.

In addition to resolving linguistic uncertainty, the first round provided information on the utility of the interventions themselves. We initially requested results for reopening after declining to 1% of peak. Round 1 results suggested this condition would rarely, if ever, be met, so that results for this intervention were not meaningfully different from those of the closed intervention, and thus we altered the intervention to trigger at 5% of peak instead. Typically, such changes in interventions would be made in consultation with decision makers (as part of Fig. S1, loop A).

Deliberately, consensus on scientific uncertainty was not required. In fact, model results were presented anonymously to reduce the pressure to conform to other groups' expectations and hence to avoid 'groupthink,' and other cognitive biases, engendering a more comprehensive expression of legitimate scientific uncertainty. We thus encouraged modeling groups to adjust their models to reflect unknown aspects of the transmission and intervention implementation process to more fully express genuine scientific and logistical uncertainty. A fuller expression of uncertainty, captured in individual models and the aggregate, allows for more robust risk

quantification. If for example, a local official or hospital administrator had to rely on only a single model to estimate the exceedance risk for hospital bed capacity, they might mis-estimate the risk and possibly over- or under-prepare.

Due to the opposing effects of decreasing linguistic uncertainty and maintaining or increasing expression of scientific uncertainty, it was difficult to draw conclusions about the source of model-level changes in expressions of uncertainty between rounds 1 and 2. To begin to assess model-level changes, we compared the lengths of inter-quartile ranges (IQRs) (Figs. S13-S15) within groups by round as well as the ratio of IQR length between each model and the corresponding aggregate distribution. The clearest examples of model incorporation of additional scientific uncertainty in round 2 were the models that provided point estimates in round 1 (length of IQR = 0) that subsequently expanded these estimates to distributions in round 2. Requiring distributions rather than point estimates necessarily increased the degree of expressed uncertainty. However, even in these models, we observed decreases in uncertainty (presumably in linguistic uncertainty) as the point estimates account for the majority of outliers in round 1 (Fig. S13).

For each objective-intervention pair considered in both rounds, the length of the aggregate IQR was greater than the median length of the corresponding model IQRs (Fig. S14). The degree of uncertainty (as measured by IQR lengths) for the majority of models increased towards that of the corresponding objective-intervention aggregate distribution from round 1 to round 2 (see the clustering of points near the orange dashed line in Fig. S14 round 2). Implementation of the open and closed interventions did not rely on a definition of "peak". In Fig. S15, we observed that the ratio of IQRs (IQR(model)/IQR(aggregate)) between rounds tended to be closer to one than the 2-week intervention, which required a definition of peak (Fig. S15). We also note that decreases in the IQR length for the aggregate distribution were observed for all objectives in the 2-week scenario (i.e. aggregate ratio of $IQR_{R2}/IQR_{R1} <1$). Changes observed in the open scenario (cumulative infections, cumulative deaths, and peak hospitalization ratios observed are 1.20, 1.02, and 0.949 respectively) were moderate compared to those in the closed scenario (cumulative infections, cumulative deaths, and peak hospitalization ratios observed are 1.93, 1.92, and 1.54 respectively). Note that an analogous comparison for the alternative peak intervention was not possible, given the change from 1-percent of the peak to 5-percent of the peak between rounds.

<u>Tradeoff between public health and economic objectives</u>

Balancing public health outcomes and economic considerations is an important aspect of pandemic decision making, but needs a more nuanced treatment, particularly on the economic side. If we compare each of our four interventions to a hypothetical "no disease" scenario, we can identify four broad groups of costs from multiple perspectives:

(a) financial costs caused by the disease itself (e.g., reduced economic output due to absence from work due either to illness or voluntary isolation, lower productivity at work or when

working from home, direct and indirect costs of medical treatment, or costs associated with funerals);

(b) non-financial costs caused by the disease itself (e.g., mortality, morbidity, long-term health impacts);

c) financial costs caused by the strategic response to the disease (e.g., reduced economic output due to a lockdown, costs of monitoring and enforcing a lockdown or of quarantining incoming travelers) or by individual responses that go beyond local policy; and

(d) non-financial costs caused by strategic or personal responses (e.g., mental health challenges due to isolation and stress, relationship stressors and breakdowns, family violence, reduced access to opportunities for recreation).

The magnitudes of these costs will depend on a wealth of factors, potentially including behavioral factors (e.g., forgoing preventive medical care such as routine childhood vaccinations), economic factors (e.g., poverty causing a need to work despite disease risks), and policy factors (e.g., constraints such as lockdowns). There may also be direct or indirect economic benefits associated with mitigation activities. For example, some firms have found that having staff work from home can increase efficiency and reduce operational costs (9), which could have ongoing benefits. Air pollution was reduced by reduced transport during lockdowns. The successful use of online video software to hold meetings including people from different locations is likely to result in a greater reliance on that approach post-pandemic, leading to reduced costs of travel, accommodation and lost work time and reduced emissions of greenhouse gases. The likelihood that people will adapt creatively to the constraints imposed by a pandemic or by a management strategy increases the difficulty of estimating the costs, because the nature and success of such adaptations are somewhat unpredictable.

There may be important dynamic trade-offs in the benefits and costs that arise. For example, in some circumstances it may be worth incurring higher financial costs in the short term (a more extreme lockdown) in order to achieve a more rapid opening up of the economy following successful containment of the disease. New Zealand provides an example where this strategy was successfully followed.

In principle, the optimal strategy would be that which minimizes the sum of all these costs, less any benefits. Our analysis does not quantify all of these costs and benefits but does provide evidence that could be used as key inputs to a comprehensive economic analysis. This analysis also starkly illustrates the tensions between economic and public health goals seen worldwide and suggests that strategies that only consider the timing of re-opening, or focus on a single type of intervention, may not be nuanced enough to manage these trade-offs. Feedback to decision makers from this process may lead to refined, possibly multi-criteria, objectives (via loop A in Fig. S1).

Insights from individual models

The median probability of an outbreak increased to 100% for all intervention scenarios other than closed. Even relatively stringent re-opening guidelines were insufficient to guarantee success; complete cessation of community spread of the virus was unlikely even with long-term non-essential workplace closure, i.e., non-essential workplace closures alone would be insufficient to manage COVID-19 at a county- level). Either additional stay-at-home orders would be required, or other non-pharmaceutical interventions (e.g., testing, contact tracing and

isolation, or wearing masks) or pharmaceutical interventions (e.g., vaccination) would be needed to stop transmission while allowing workplace re-opening.

Three models ranked the closed and 5-percent interventions as identical for several metrics; 6 groups reported that for at least some simulations the 5-percent reopening criterion was never met in the 6-month period. Two models ranked the 5-percent intervention as better than the closed intervention based on the medians of health outcomes; both models had wide priors on parameters governing compliance with interventions. Three of the 17 models ranked the 5-percent intervention worse than the 2-week intervention for public health measures (Fig. S12A), a result that was driven by different timing in the triggering of re-opening (Fig. S5). Another notable result is that the ranking of the 2-week intervention for the peak hospitalization metric spanned the gamut from worst rank (in submission M) to first-tied rank (in submission A) (Fig. 1). Otherwise, rankings were remarkably consistent overall.

Disagreements between models, or between models and the aggregate, were examined retrospectively, and include a range of reasons: genuine scientific disagreement about processes to include in the model given the massive uncertainty about SARS-CoV-2 at the time; stochasticity (especially in the case of very close or "tied" results); differences in calibration approaches; residual linguistic uncertainty (language uncertainty was drastically reduced but not completely eliminated by group discussions); inclusion of assumptions groups would later choose to revise if more time was available (especially with the benefit of hindsight).

Comparison of county death and case data with aggregate model results

The modeling exercise was motivated by a U.S. county representative of mid-sized counties with populations of approximately 100,000 people, with limited mobility and stay at home orders in place until at least May 15, 2020. Here, we compare aggregate model results with reported data from U.S. counties meeting the target county description.

We first selected the 98 U.S. counties with population sizes between 90,000 and 110,000 using data from the Johns Hopkins University COVID-19 dashboard (9). From this subset, we then selected counties with stay at home orders in place until at least May 15, 2020 (data from (10–12)), and changes in mobility in line with stay at home orders, i.e., less than 50% increase from baseline retail mobility, less than 25% increase in baseline work mobility, and less than 5% decline from baseline residential mobility (data from (13)). This resulted in a subset of 85 counties. Finally, from this subset, we identified 18 counties implementing a fully 'closed' intervention (with stay at home orders in place and unmodified from May 15, 2020 to November 15, 2020 and mobility patterns suggesting those orders were followed). Relaxing the definition of 'closed' to counties with any stay at home orders in place (including unmodified, modified, partial or stay safe at home orders) from May 15, 2020 to November 15, 2020, the subset of counties considered to be 'closed' increases to 84 (data from (14)). One county was found to be fully open during this period, and it was not possible to determine if any counties implemented the '2-week' or '5-percent' interventions. We compared aggregate cumulative reported deaths and cases with modeled cumulative deaths and infections (all COVID-19 deaths and infections, both reported and undetected) under the closed intervention for the 18 counties following the

‘closed’ intervention according to the strict definition of closed (including only full stay at home orders) and the 84 counties following the ‘closed’ intervention according to the relaxed definition of closed (including full and partial stay at home orders). Cumulative reported deaths and cases for the two groups of counties under the closed intervention were summarized in 100 quantiles, the same format requested from model groups (See Figs. S16 - S17, below).

Note that we are comparing *reported* deaths and cases (from data) with *all* COVID-19 deaths and cases from model results (which captures reported and undetected infections). We did not assume a reporting or detection rate, but perforce expect a higher number of model-predicted cumulative deaths and cases. Crucially, our results represent the realization of one pandemic across multiple counties in comparison to multiple model realizations across a wide range of uncertainty. Thus, the model uncertainty will necessarily be higher than the observed uncertainty. The model mean will likely also be higher, as the right-skewed uncertainty will increase the mean.

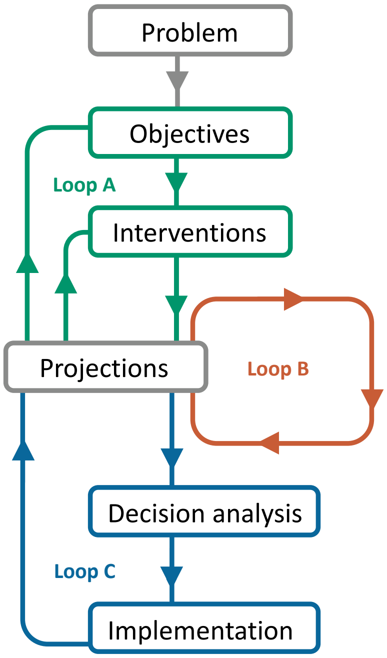

Fig. S1: Multiple Models for Outbreak Decision Support (MMODS) framework, specifically for the elicitation in this project. The **Problem** is the decision context faced by state and local officials regarding local guidance and regulations concerning the operation of non-essential workplaces, in the face of the COVID-19 pandemic during the period May 15 to November 15, 2020. The 5 **Objectives** addressed were to minimize: (1) cumulative infected individuals, (2) cumulative COVID-related deaths, (3) peak hospitalizations, (4) probability of a new local outbreak (more than 10 new reported cases/day), and (5) total days workplaces closed, all over the period May 15 to November 15. The four **Interventions** focused on strategies for re-opening non-essential workplaces, while assuming all schools remaining closed, between May 15 and November 15, 2020: (1) continue with current non-essential workplace closures, (2) open non-essential workplaces when the number of new daily cases is at 5% of peak, (3) open non-

essential workplaces 2 weeks after peak, and (4) immediately relax all current restrictions on non-essential workplaces. Loop B coordinates modeling groups to reduce bias and linguistic uncertainty. First, loop B involves independent (round 1) model **Projections** of all objective-interaction combinations. A structured, facilitated group discussion reduces unwanted uncertainty and also prompts information on additional sources of data used, methods used to incorporate uncertainty, and assumptions made by individual groups, so that the whole collaborative can improve their models. Retention of the remaining model differences allows for a more comprehensive expression of legitimate scientific uncertainty; consensus is not required. Modelling groups then provide updated (round 2) model projections. Loop A provides an opportunity for model groups to interact with decision makers to clarify or update objectives or interventions, i.e., to reduce linguistic uncertainty. **Decision Analysis** is used to aggregate and analyze the model outputs to rank interventions. If decisions are implemented, then there is also an opportunity for modeling teams to learn from **Implementation** data and results (loop C)

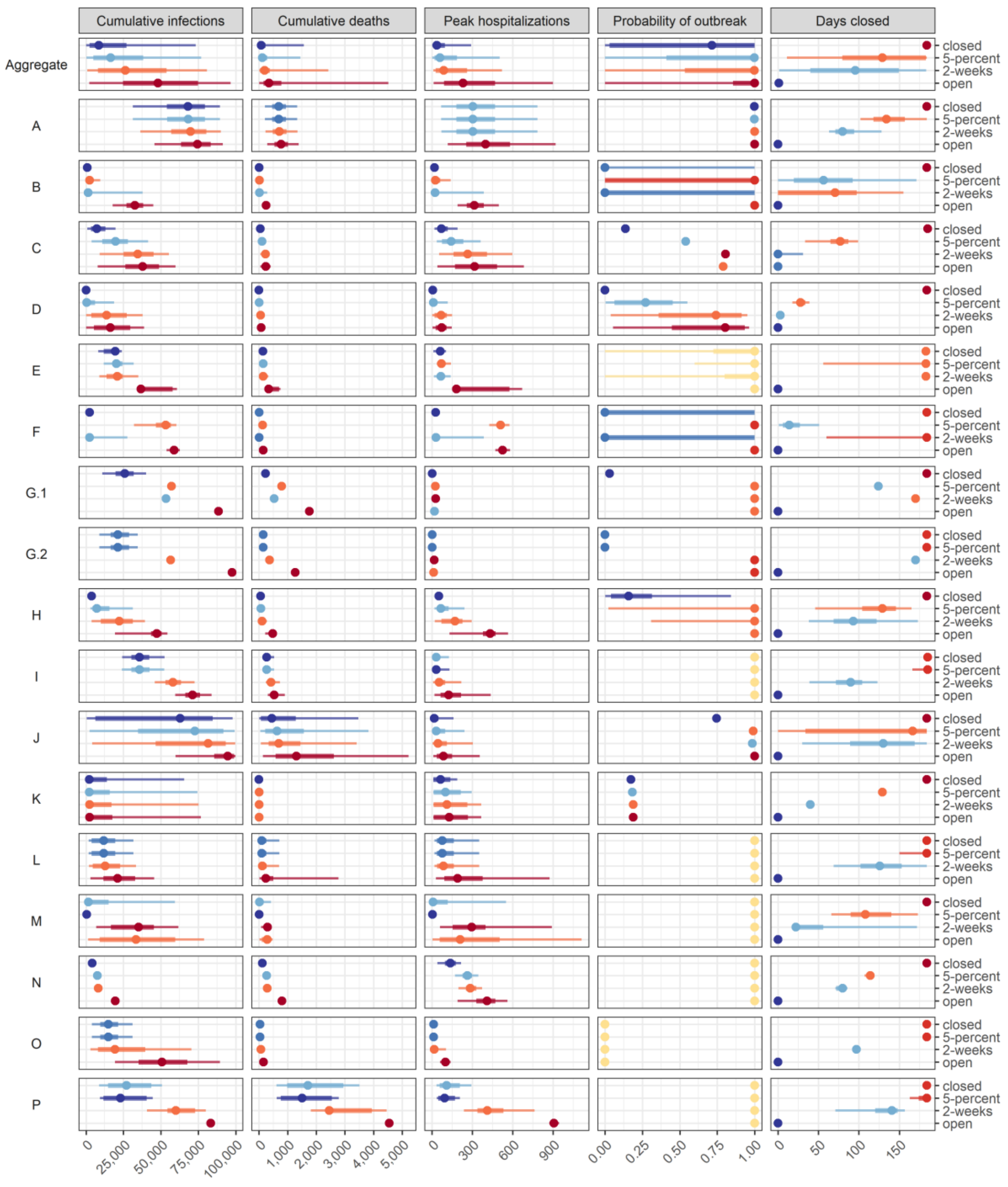

Fig. S2: Model results for each target objective and intervention scenario pair organized by model. Medians, 50% prediction intervals (PIs), and 90% PIs are indicated as points, thick lines, and thin lines, respectively. Colors denote ranking of each intervention by model for a single objective, where dark blue signifies the lowest value (best performance) and dark red signifies the highest value (worst performance). Ties in ranks are colored as intermediate values. A tie between ranks 1 and 2 and ranks 3 and 4 are shown as an intermediate blue and red, respectively; yellow indicates a tie in ranks across all interventions. Each group is assigned a random, unique identification letter that is specified on the vertical axis.

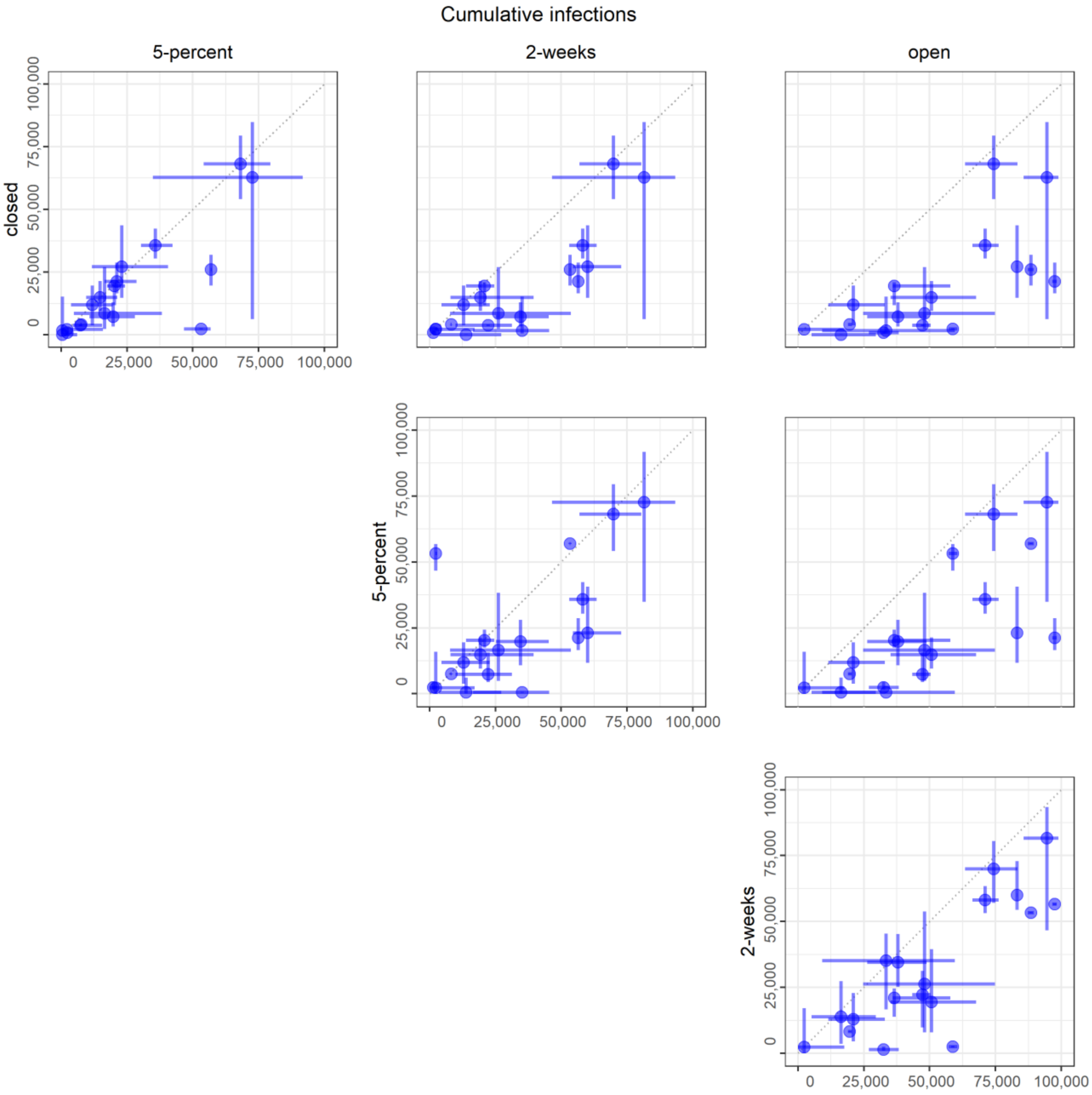

Fig. S3: Cumulative infections. Medians (points) and 50% PIs (lines) displayed pairwise by intervention scenario. Each point represents one model.

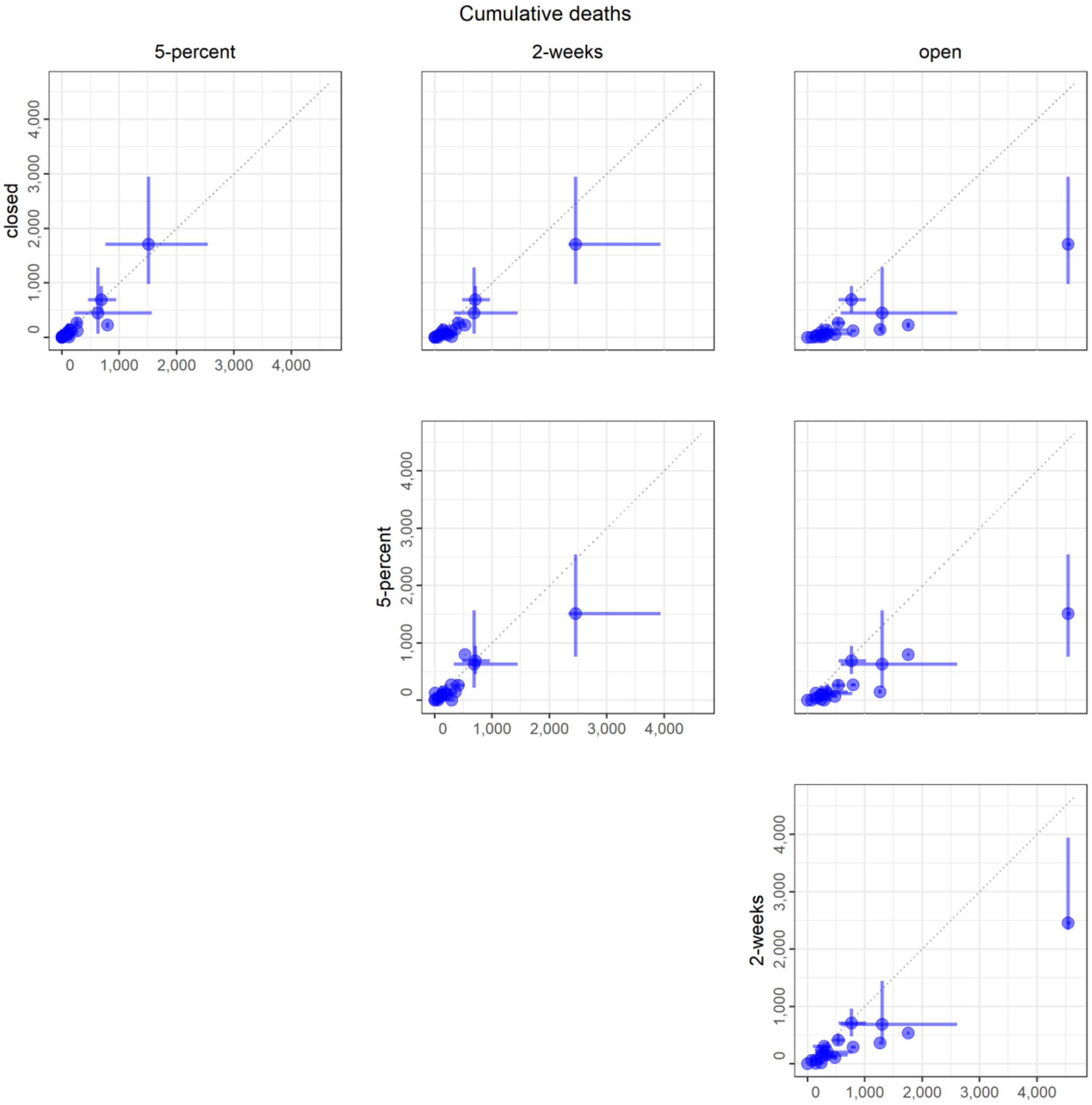

Fig. S4: Cumulative deaths. Medians (points) and 50% PIs (lines) displayed pairwise by intervention scenario. Each point represents one model.

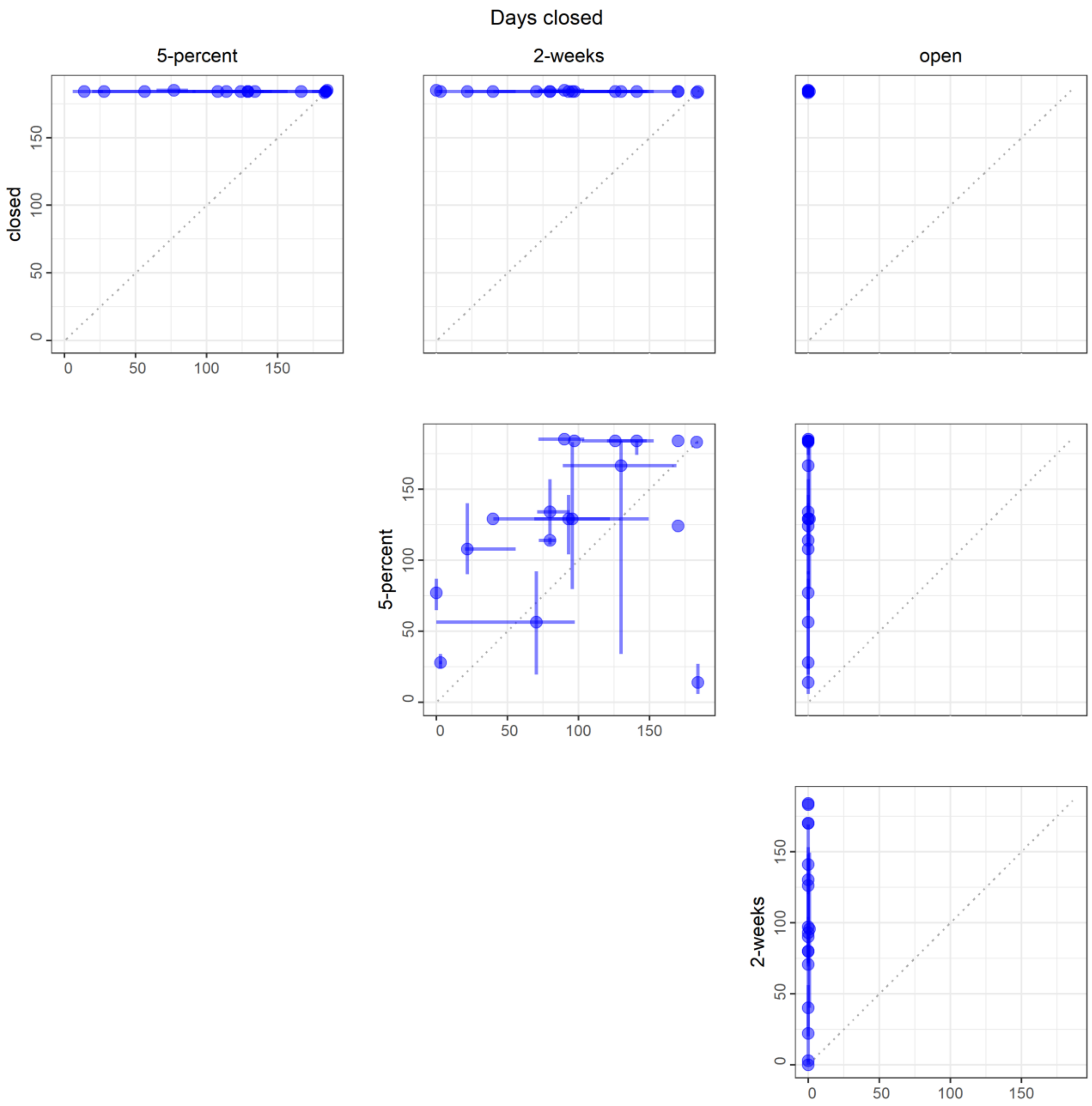

Fig. S5: Days closed for non-essential workplaces. Medians (points) and 50% PIs (lines) displayed pairwise by intervention scenario. Each point represents one model.

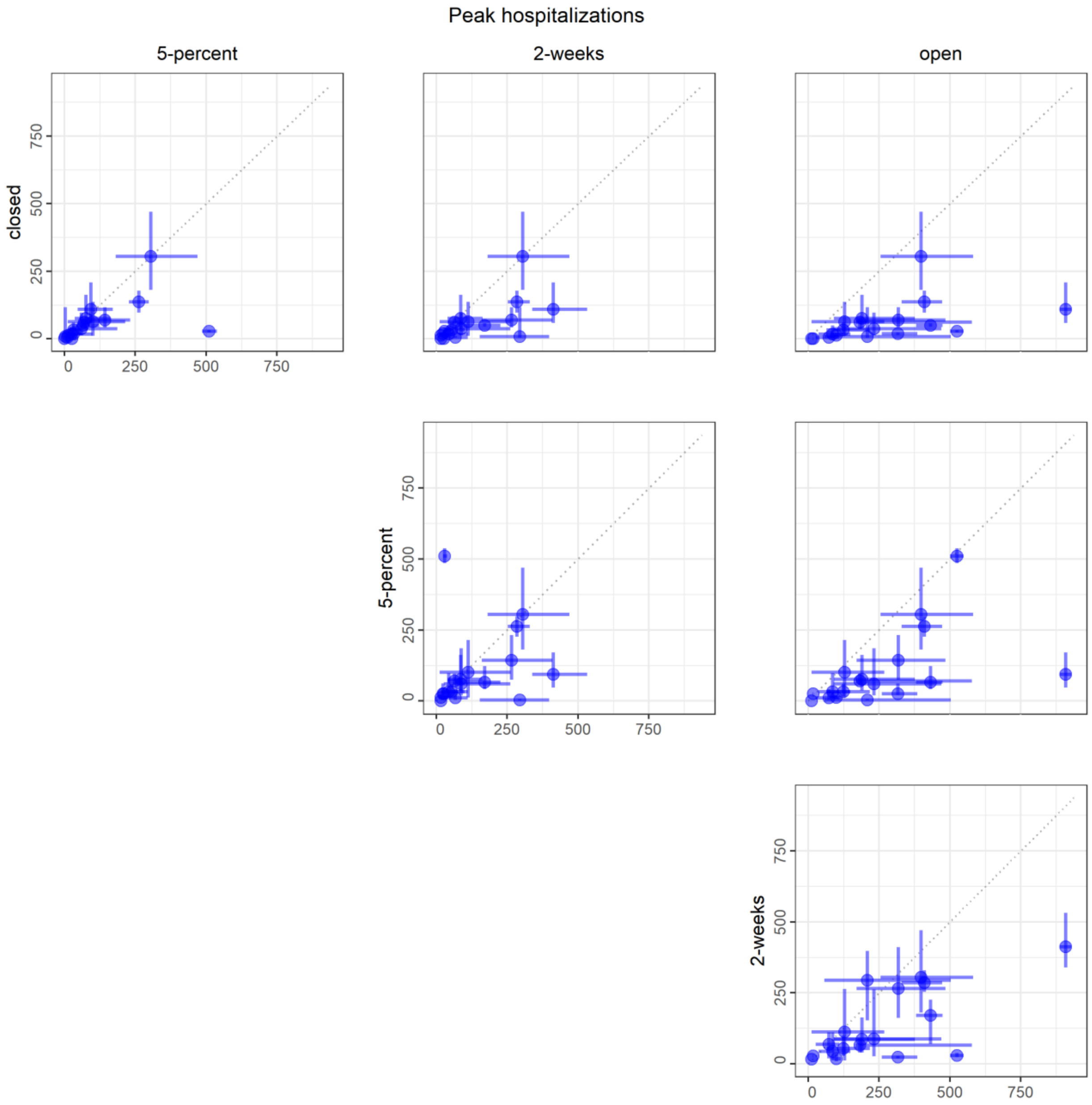

Fig. S6: Peak number of hospitalized cases. Medians (points) and 50% PIs (lines) displayed pairwise by intervention scenario. Each point represents one model.

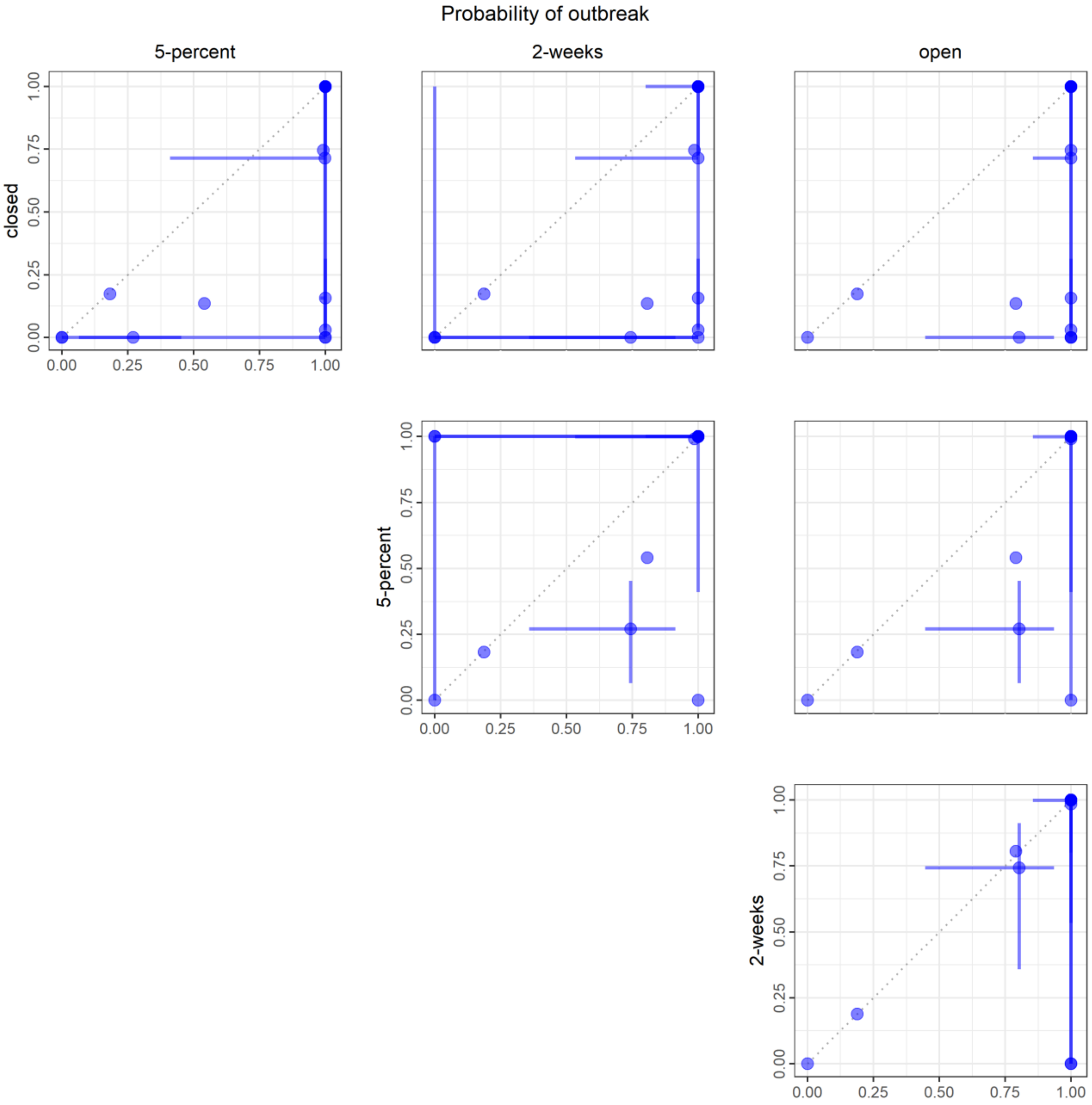

Fig. S7: Probability of outbreak. Median (points) and 50% PI (lines) displayed pairwise by intervention scenario. Each point represents one model.

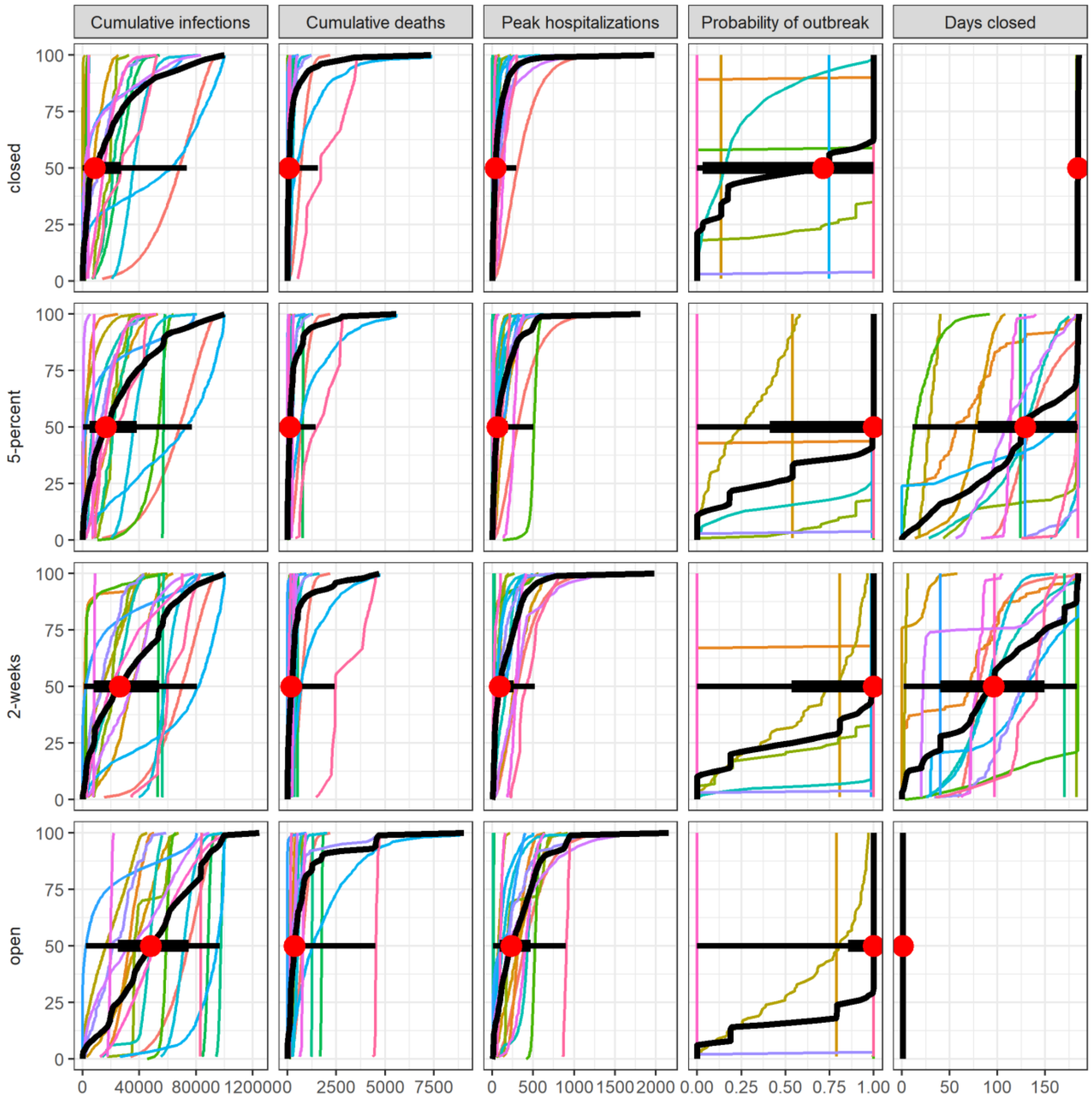

Fig. S8: Cumulative distribution functions (CDFs) across models and for the aggregate. Each colored line shows the quantile distribution for a single model. Each aggregate CDF is shown in black with medians, 50% PIs, and 90% PIs indicated as red points, thick lines, and thin lines respectively.

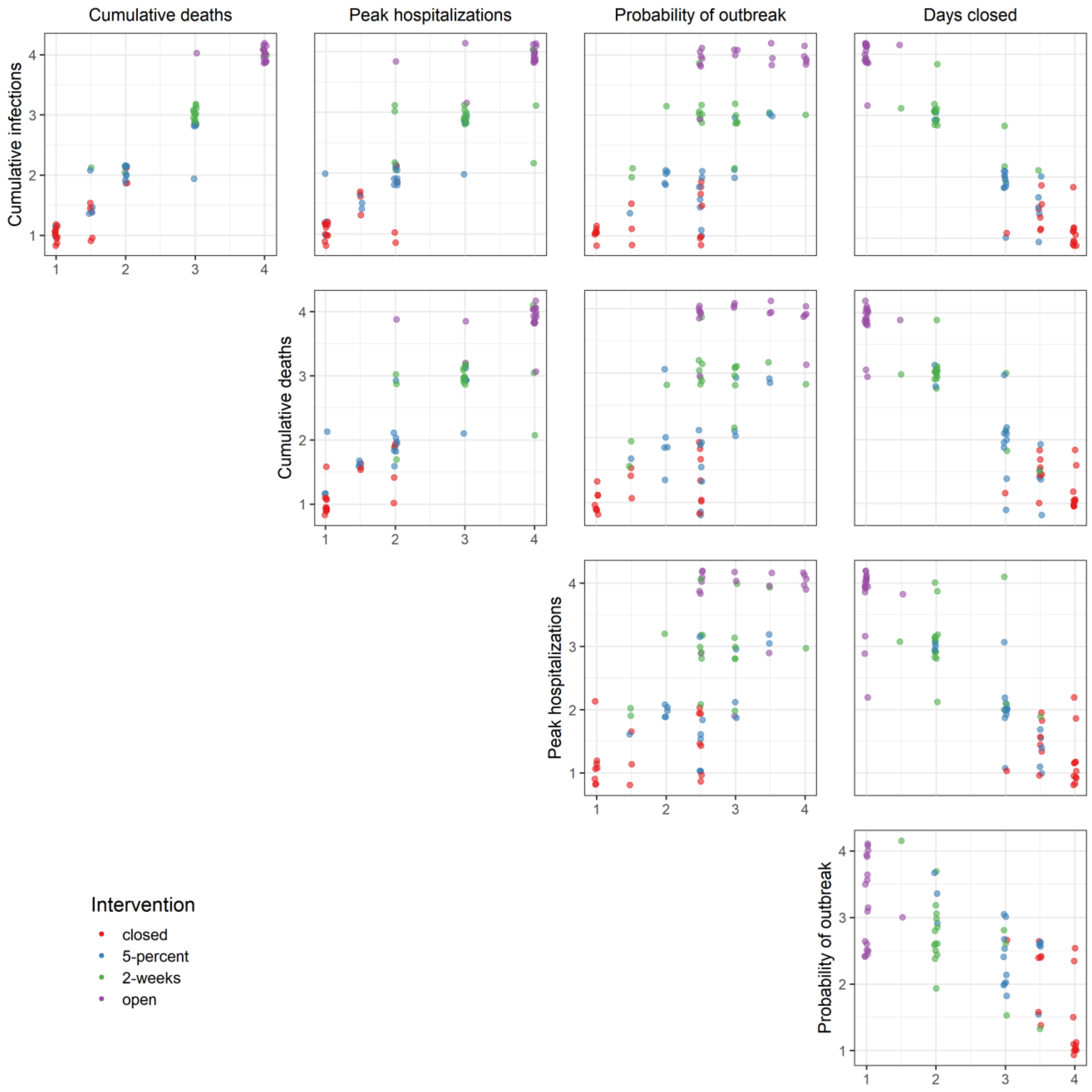

Fig. S9: Scatter plots of intervention ranks for a given pair of objectives. Rank ties are shown as intermediate numerical values (e.g. a tie between 1 and 2 is shown as 1.5). For visual clarity, shaded points are jittered around the discrete rank values.

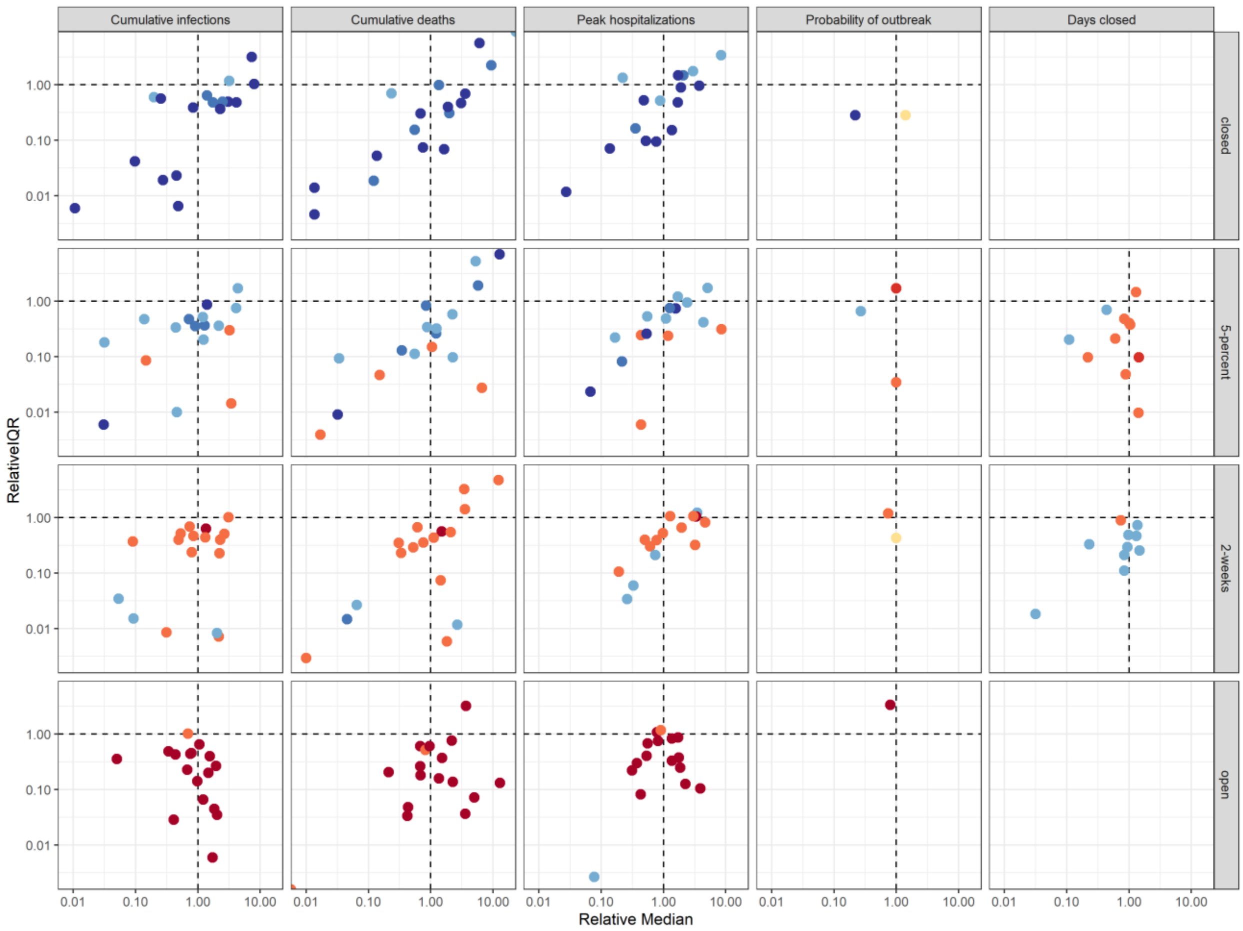

Fig. S10. Comparison of individual model results to aggregate results. The y-axis shows the relative interquartile range (IQR)—the ratio of an individual model's IQR to the aggregate IQR. The x-axis shows the ratio of an individual model's median to the aggregate median. Both axes are presented on a log scale. Colors denote ranking of each intervention by models, where dark blue signifies the lowest value (best performance) and dark red signifies the highest value (worst performance). Ties between ranks 1 and 2 and ranks 3 and 4 are shown as an intermediate blue and red, respectively; yellow indicates a tie in ranks across all interventions.

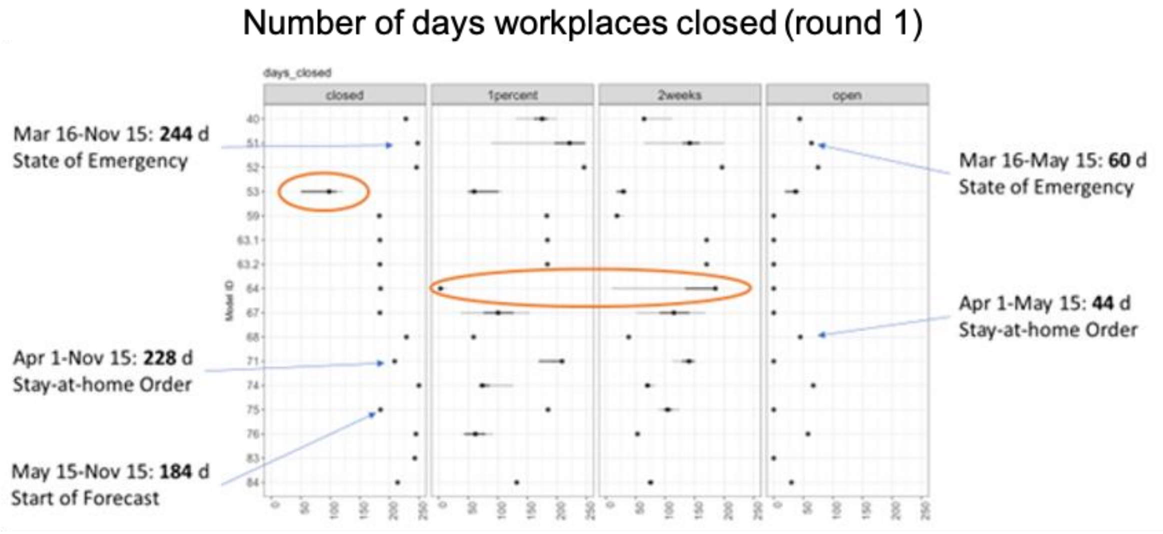

Fig. S11: **Resolution of linguistic uncertainty in the discussion following round 1 of modeling (note that model IDs changed between rounds).** Presentation slide from the group discussion after round 1 demonstrating linguistic uncertainty about the number of days non-essential workplaces are closed. Ovals highlight points of discussion about different ways of capturing uncertainty for days workplaces are closed and unusual results about intermediate interventions. See main text Fig. 2 for days of non-essential workplace closure results from round 1 versus round 2.

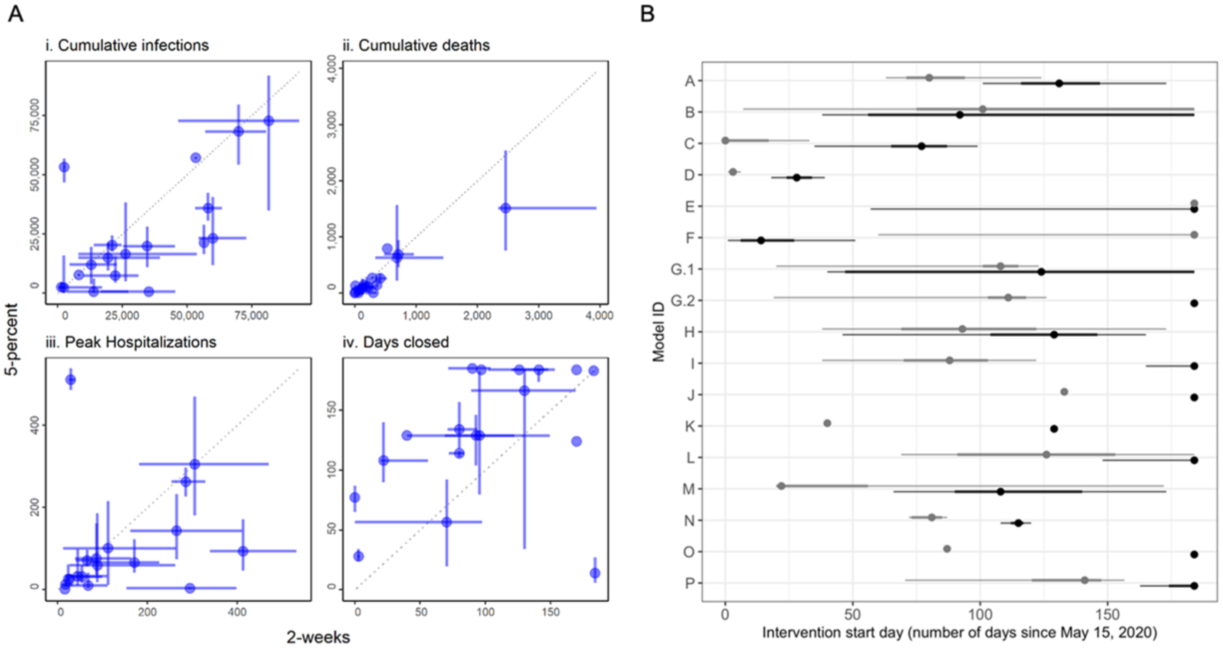

Fig. S12. Comparison between the 2-week and 5-percent interventions. A) Medians (points) and 50% PIs (lines) displayed pairwise by intervention and for the following objectives: i) cumulative infections, ii) cumulative deaths, iii) peak hospitalizations, and iv) days closed for each model. **B)** Comparison of intervention start dates for 2-week (grey) vs. 5-percent (black) interventions for each model, where the start date is computed as the number of days from May 15 until the intervention is enacted. Intervention start times of 184 days indicate that the intervention was never triggered in that model. All plots display median (points) and 5th to 95th quantiles (lines) for each intervention. The 2-week intervention trigger to open is the first day for which the 7-day trailing moving average of the number of new daily reported cases has been lower than the maximum for at least 14 days, and has shown a day-to-day decline in smoothed case data for at least 10 of the last 14 days (or, there have been 7 days without any new cases). The 5-percent intervention trigger to open is the first day for which the 7-day trailing moving average of the number of new daily reported cases drops below 5% of the maximum.

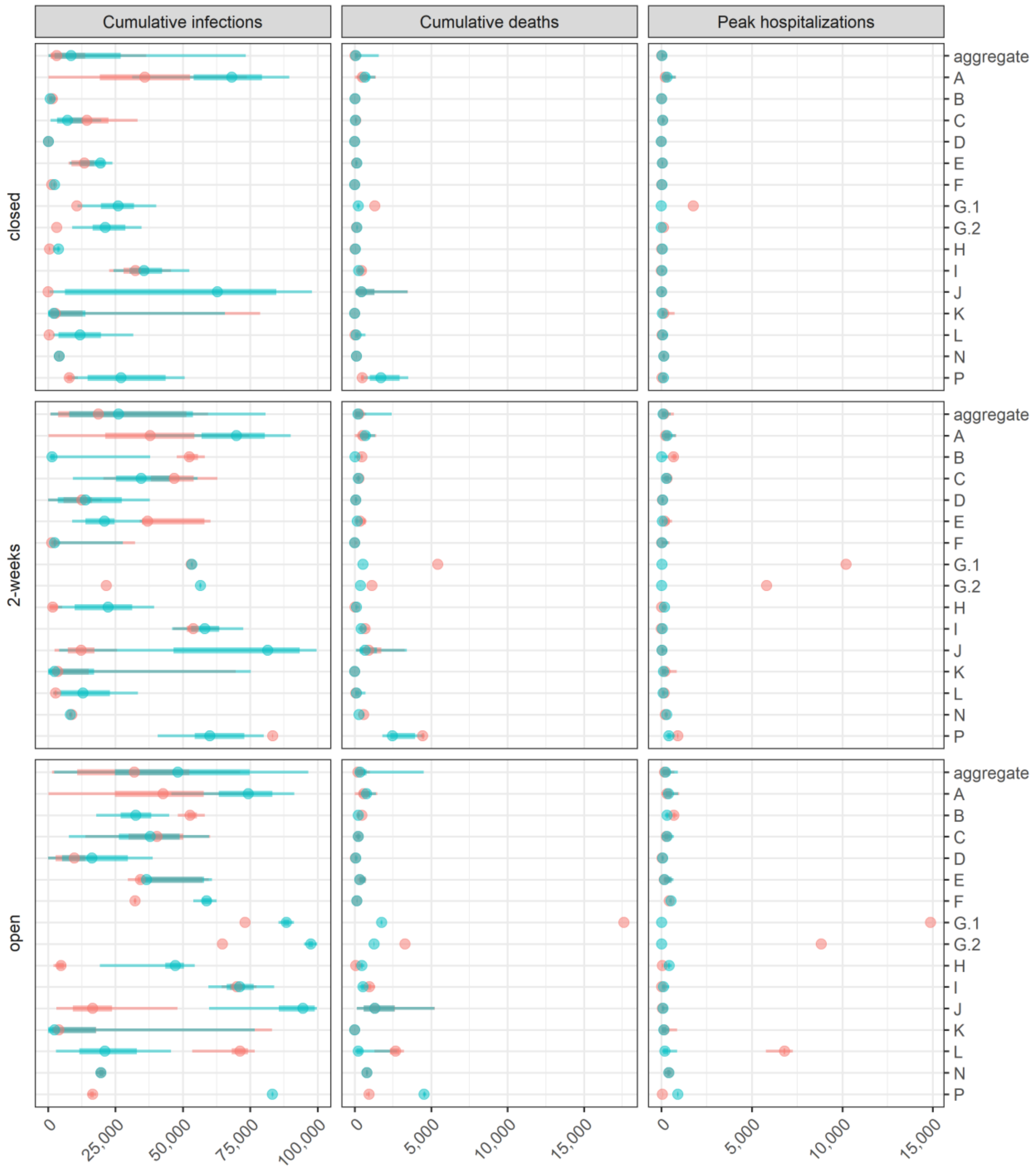

Fig. S13: Team and aggregate values for each intervention and objective pair. Round 1 and round 2 results displayed in red and blue respectively. Since the 1-percent intervention from round 1 was updated to a 5-percent intervention in round 2, results for these interventions have been omitted from this comparison. Also note that two models were excluded from this analysis, as they submitted incomplete results in round 1. After the discussion between rounds 1 and 2, these groups were able to provide complete and comparable results. Additionally, in at least one case, some of the differences can be attributed to model error fixes between rounds.

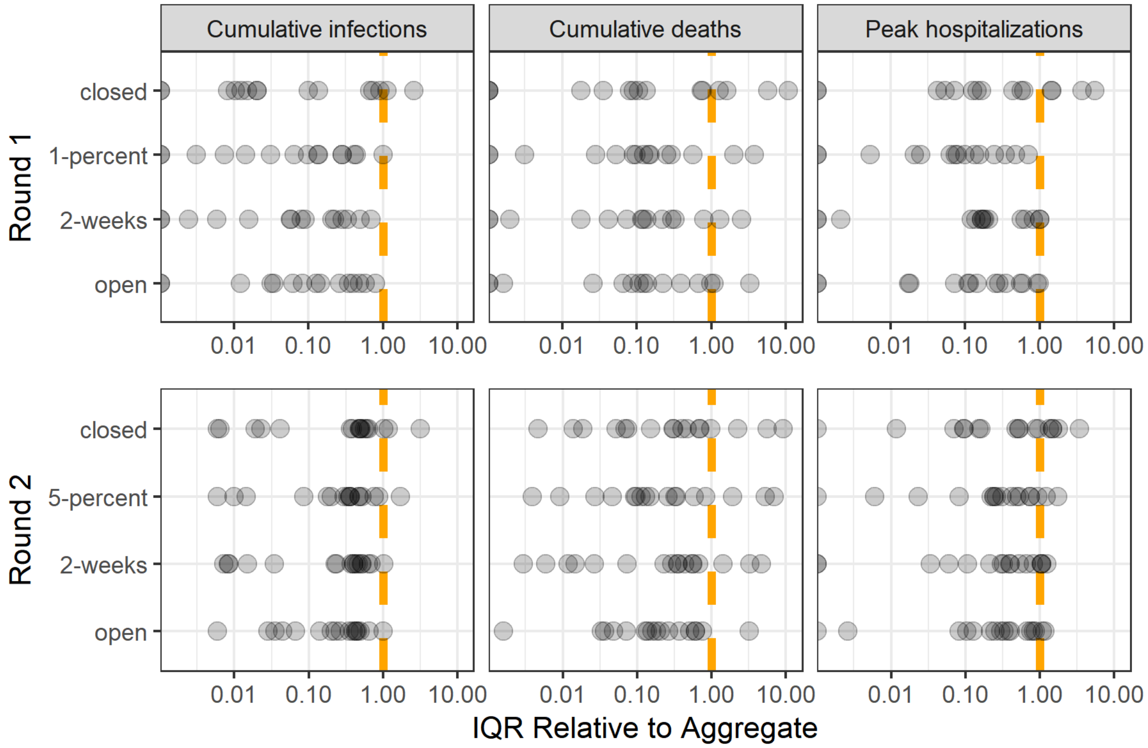

Fig. S14: Comparison between model-specific IQR lengths and the length of the IQR for the aggregate distribution (i.e. length(IQR_team)/length(IQR_aggregate)) shown on a logarithmic scale. Results are grouped by round, intervention, and objective. Round is indicated on the left axis. Columns indicate the objective and rows indicate the intervention. The dashed orange line highlights the point at which there is no difference between the model-specific IQR lengths between rounds 1 and 2 (points to the left indicate a model IQR less than that of the corresponding aggregate and *vice versa* for points to the right).

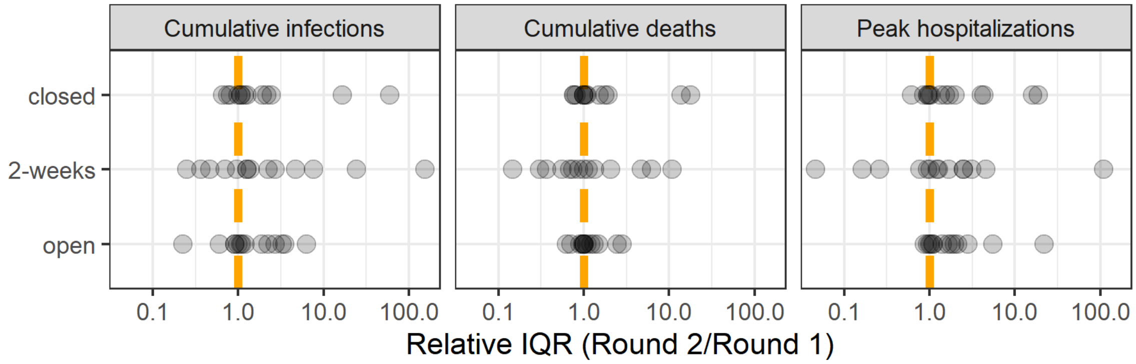

Fig. S15: Round comparison of IQR length by team, calculated as the ratio of the length of IQRs between rounds 1 and 2 (i.e. length(IQR_{R2}) / length(IQR_{R1})) shown on a logarithmic scale. Note that in the first round, two models (G.1 and G.2) submitted point estimates for each intervention and metric. Since point estimates are such that length(IQR) = 0, the relative IQR (compared to round 1) is infinity and thus not shown here. Similarly, there is not a point representing cumulative deaths in the closed scenario for group K since the corresponding length(IQR) = 0. Because the 1-percent intervention from round 1 was changed to a 5-percent intervention in round 2, the corresponding results have been omitted from this comparison. The dashed orange line highlights the point at which there is no difference between the model-specific IQR lengths between rounds 1 and 2 (points to the left indicate a lower R2 IQR than that of the corresponding group's R1 submission, and vice versa for points to the right).

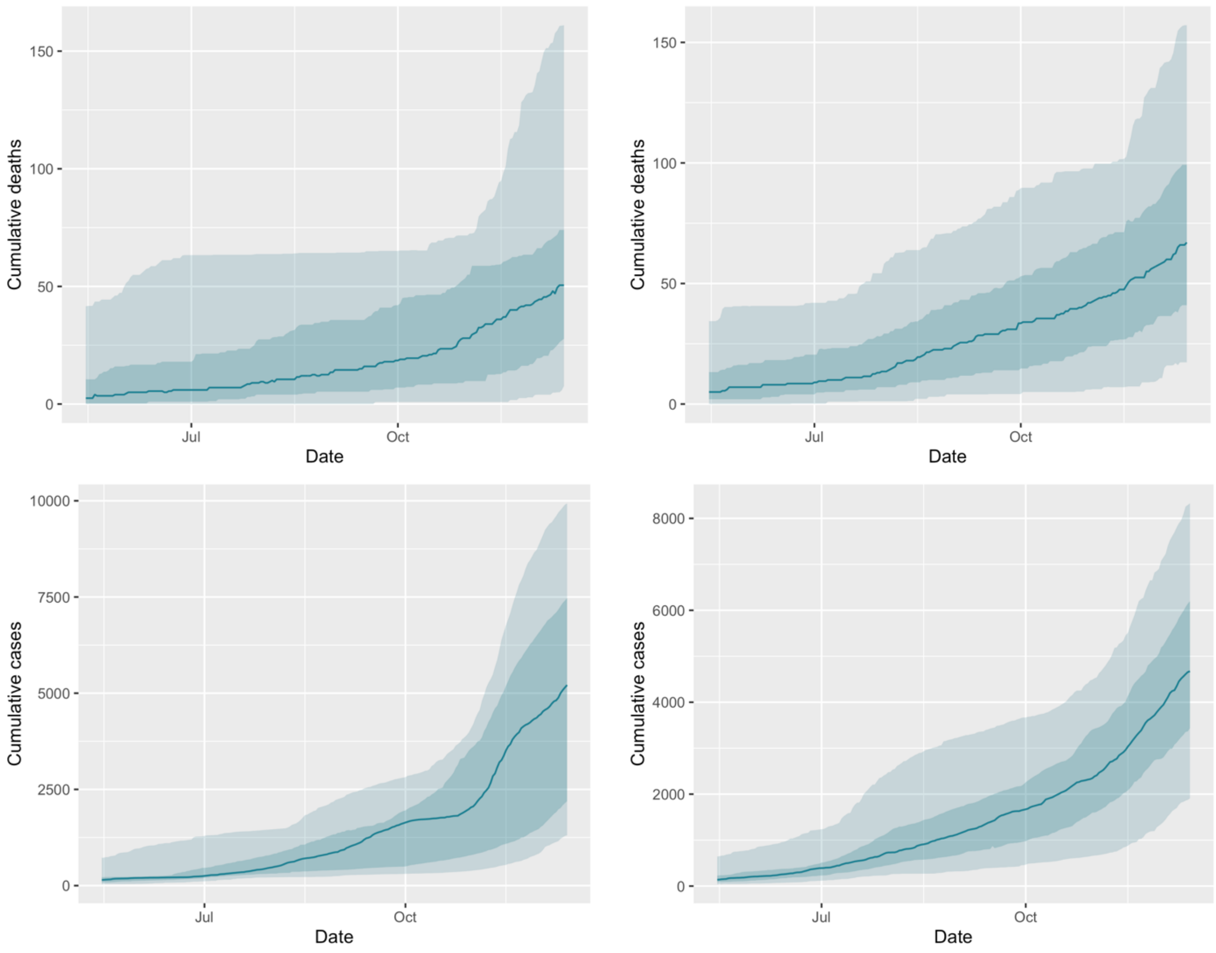

Fig. S16: Summary of cumulative reported deaths (top) and cases (bottom) for counties similar to the model context and following the closed intervention i.e., with full stay at home orders in place (left) or full and partial stay at home orders in place (right). Median reported cumulative deaths (solid line), 50% IQR (darker shaded area), and 90% IQR (lighter shaded area) for the subset of 18 counties with full stay at home orders in place and the subset of 84 counties with full or partial stay at home orders in place from May 15 to November 15, 2020.

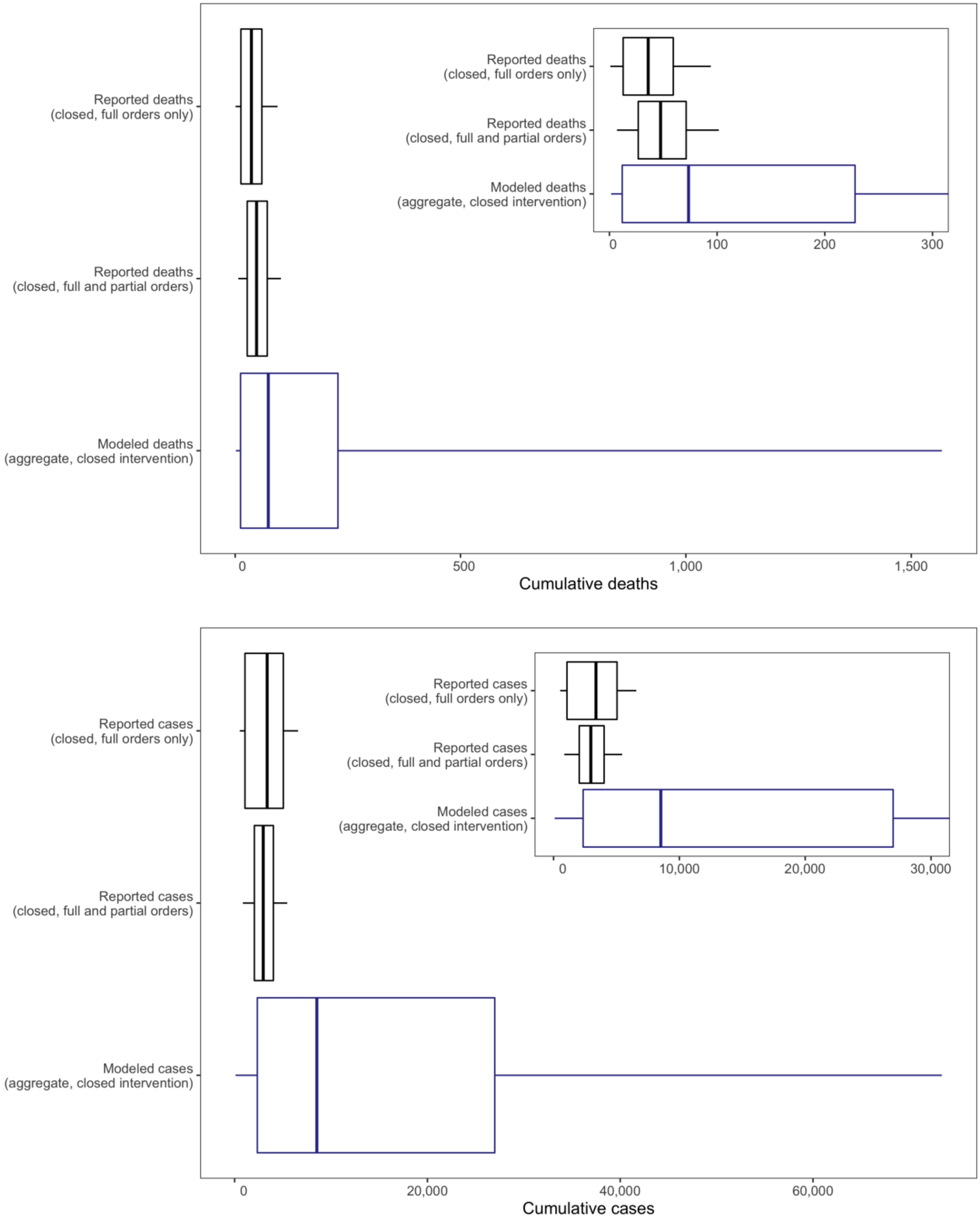

Fig. S17: Comparison of aggregate reported county death and case data to modeled deaths and infections for the closed intervention according to two definitions of closed (full stay at home orders in place or full and partial stay at home orders in place). Top: Boxplot of

cumulative reported deaths from 18 and 84 U.S. counties with full or full and partial stay at home orders in place, respectively from May 15 to November 15 (median deaths for full orders in place: 36; 50% IQR: 13, 59; median deaths for full and partial orders in place: 48; 50% IQR: 27, 71) and model results for cumulative deaths from May 15 to November 15 under the closed intervention (median deaths: 73; 50% IQR: 12, 228). Vertical line shows median value, box shows IQR (Q25-Q75, and whiskers show Q5-Q95. Inset shows overlap of box area for the plots. **Bottom**: Cumulative reported cases from 18 and 84 U.S. counties with full or full and partial stay at home orders in place, respectively from May 15 to November 15 (median cases for full orders in place: 3374; 50% IQR: 1070, 5047; median cases for full and partial orders in place: 2964; 50% IQR: 2044, 4108) and model results for cumulative cases from May 15 to November 15 under the closed intervention (median infections: 8527; 50% IQR: 2351, 26988). Vertical line shows median value, box shows IQR (Q25-Q75, and whiskers show Q5-Q95. Inset shows overlap for the two plots.

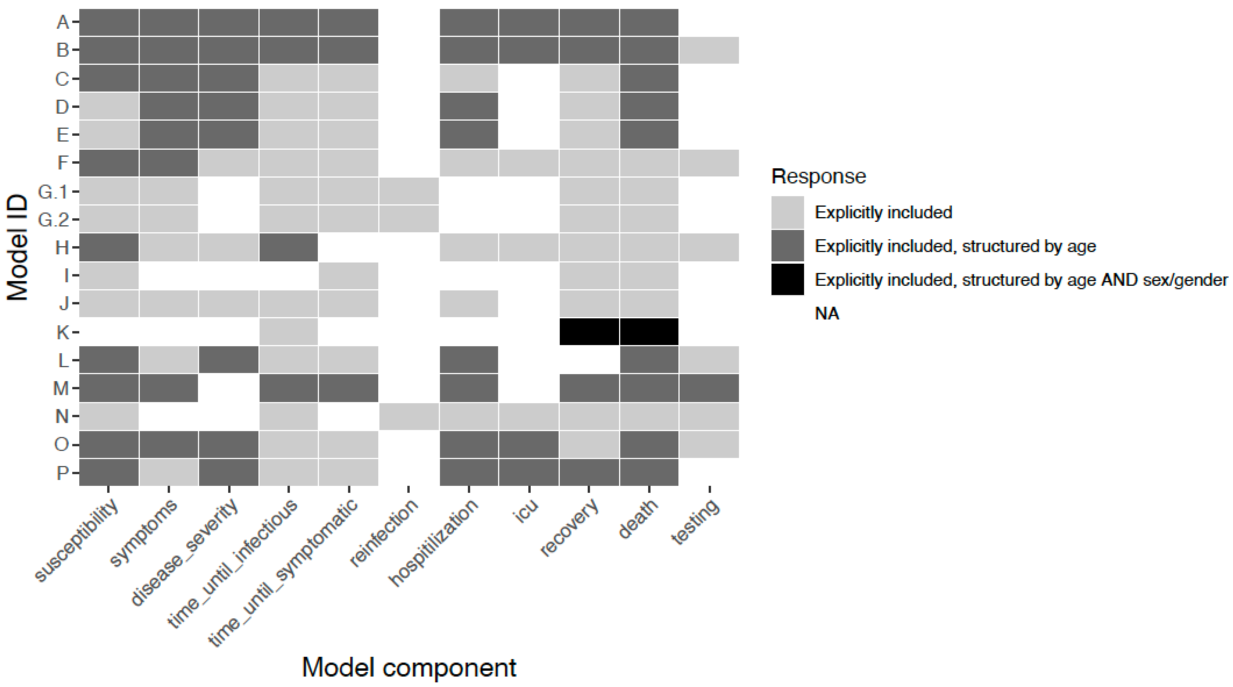

Fig. S18: Description of model components and structure by model. Participants were asked to indicate which model components were included in their model (from a given set) and whether any component was structured by age and/or gender and/or sex as part of the submission checklist. No model included any components structure by gender and/or sex. Twelve of the 17 included at least one component structured by age.

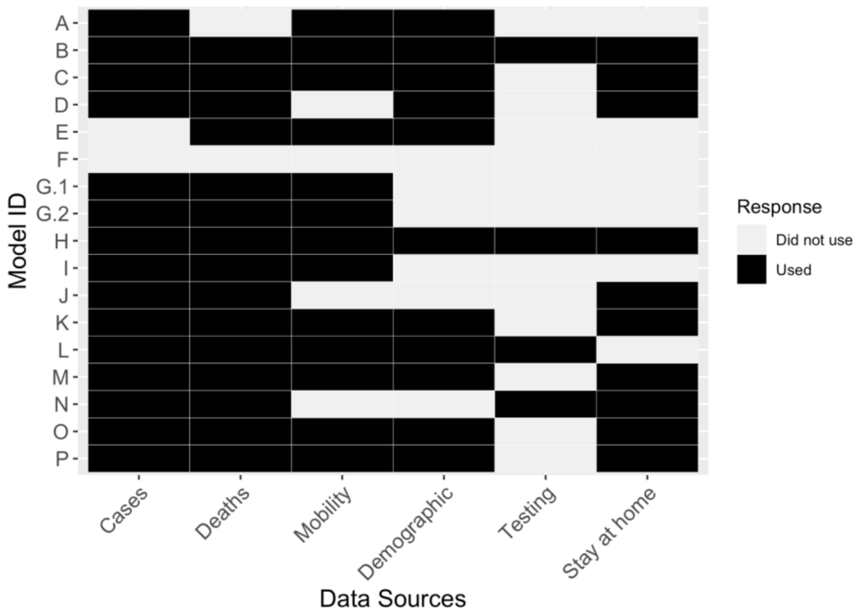

Fig. S19: Data sources used for each model. Participants were asked to indicate which of the provided datasets were used for any part of the model (e.g., for calibration, training, fitting etc.) as part of the submission checklist. All but one model used at least two of the provided data. Model F used only external data sources (provided data was used solely to better understand the intent of the exercise).

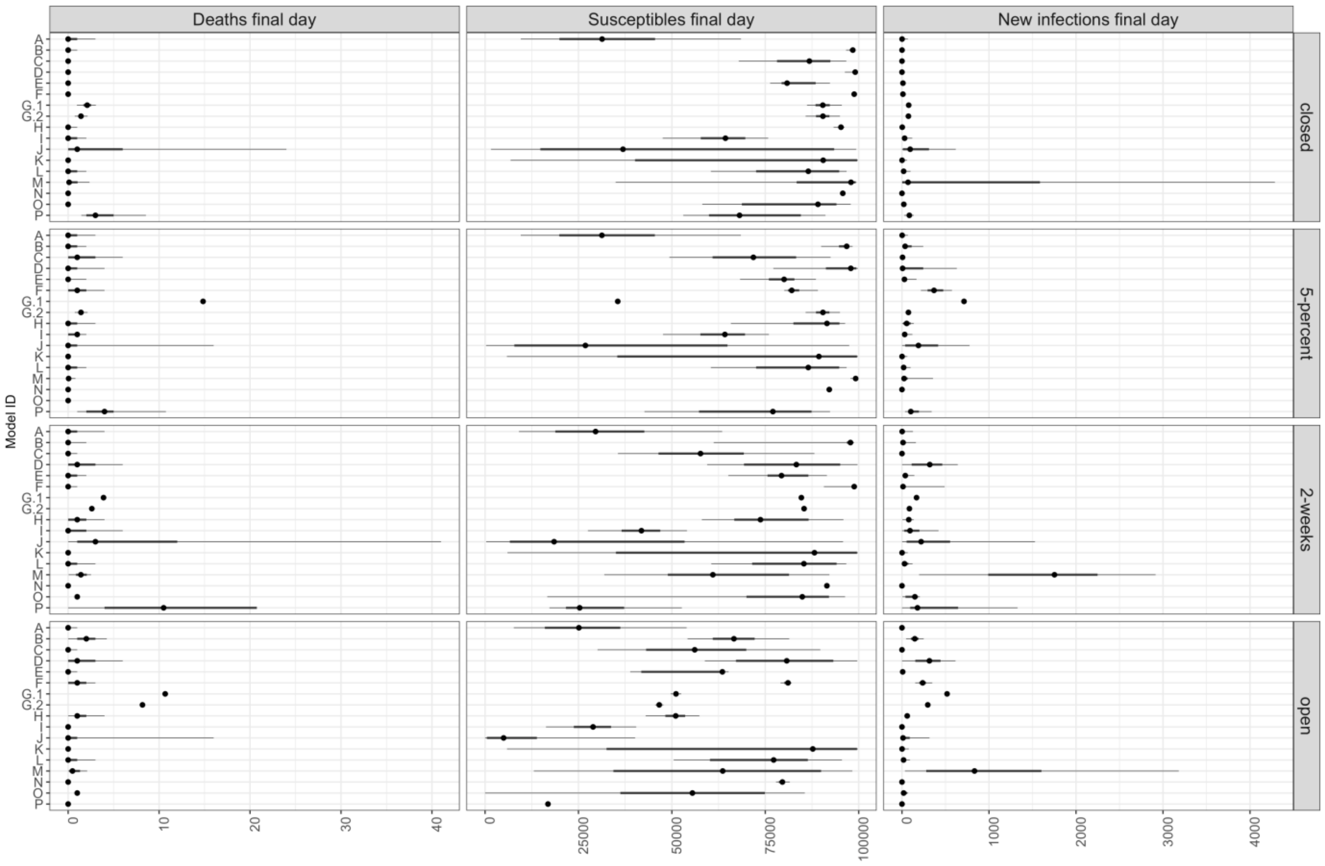

Fig. S20: Projected number of deaths, people who are susceptible, and new infections under each scenario for the final day of the forecast. Participants reported the 5th, 25th, 50th, 75th, and 95th quantiles for the number of deaths, susceptibles, and new infections on the final day (November 15, 2020) under each scenario. All models started with similar initial susceptibles.

Supplementary Tables

Table S1: Contributed model descriptions. Name, description (including links to model code where available), diagram, calibration method, other non-pharmaceutical interventions (NPIs) included in the model, additional data sources used, previous use cases for the model (both for COVID-19 in other settings and other disease systems) and references for each of the 17 models. Categories which were not relevant were excluded.

**CoMo Collaborative COVID-19 Model**	
Description	Age-structured, SEIR compartmental model with infected compartments stratified by symptoms, severity and treatment seeking and access. Code available: https://github.com/ocelhay/como
Additional NPIs included	Social distancing, Isolation (post infection), Stay-at-home (voluntary), Handwashing, Travel ban
Additional data sources used	National data on hospital, ICU, and ventilator availability; Data on U.S. household size from the American Community Survey; Data from China and New York City for healthcare parameterization; Age-structure mixing matrices for Work, School, and Home from Prem, Cook, and Jit (2017); Vital surveillances from The Novel Coronavirus Pneumonia Emergency Response Epidemiology Team (2020); NYS Governor Cuomo Daily presentation (April 23, 2020); List of countries by hospital beds (Wikipedia)
Previous use cases	RSV in Thailand
References	(15)
Co-authors	Ricardo Agus, Lisa White, Nathaniel Hupert (PI)
Acknowledgements	Wirichida Pan-Ngun, PhD, Olivier Celhay, Vruj Patel, Lior Shtayer
**Covasim**	
Description	Stochastic agent-based model, including age-structured mixing, susceptibility to infection and health outcomes; transmission networks in different social layers; variable intrahost viral dynamics; presymptomatic, symptomatic, and asymptomatic transmission; hospitalizations (regular and intensive care); and multiple non-pharmaceutical and testing interventions. Code available: https://github.com/institutefordiseasemodeling/covasim
Diagram	See (16), Fig. 1
Calibration	Parameters were calibrated by optimizing the L1 relative error norm of positive diagnoses, number of deaths, and number of tests using global optimization package, *Optuna*
Additional NPIs included	Social distancing, Isolation (post infection), and school, workplace, and community closures based on stay-at-home and state-of-emergency orders.
Previous use cases	COVID-19 in Africa, Europe, Oceania, and North America
References	(16)
Co-authors	Rafael C. Nez, Katherine Rosenfeld, Gregory R. Hart, Daniel Klein, Cliff C. Kerr (PI)
Acknowledgements	Dina Mistry, Prashanth Selvaraj, Jamie A. Cohen, Michael Famulare, Robyn M. Stuart, Romesh Abeysuriya
**EvoNet SARS2**	
Description	Stochastic, place-based model in which agents travel to different locations within the community. Agents can infect others within homes, schools, workplaces and other regular gathering spots, as well as during random walks within the community.

Calibration	Created ~1000 parameter sets each with 52 uniformly distributed parameters. For each parameter set, we considered a range of transmission probabilities. Interventions were simulated for parameter sets for which case and death counts came within range of the county data up to May 15th.
Additional NPIs included	Social distancing, Quarantine (post exposure), Isolation (post infection), Stay-at-home (voluntary), Age-specific interventions (e.g., isolation of elderly)
Previous use cases	HIV
Co-authors	John Mittler (PI)
Acknowledgements	Joshua Herbeck, James Murphy, Neil Abernethy, Sarah Stansfield, Molly Reid, Steven Goodreau
Funding	NIH grants R01AI108490 and R01 GM125440
**JHU-CDDEP Bayesian Three-stage ODE Model**	
Description	Bayesian, mechanistic ODE-based compartmental model composed of three transmission stages with varied force of infection pre-lockdown, lockdown, post-lockdown. Each stage corresponds with lockdown phases and social distancing measures that might be imposed by public health policymakers.
Calibration	Bayesian inference was conducted using MCMC-based method was used to fit the model to confirmed cases and deaths. Parameter ranges were estimated form the posterior distribution. Prior distribution was assumed to be uniformly distributed
References	(17)
Co-authors	Gary Lin, Yupeng Yang, Eili Klein
Acknowledgements	Anindya Bhaduri, Max Pinz, and the U.S. Centers for Disease Control and Prevention (CDC) Modeling in Infectious Diseases Network
**LANL1-EpiCast**	
Description	Agent based model with communities, households, and workplaces
Calibration	Transmission rates were varied in burn in period (March to May) to try to model actual county statistics. Burn in transmission rates calculated by parameter testing the model were much higher than previous experience fitting COVID-19 (0.43), and were thus scaled down to an assumptive 0.2. This is potentially due to very low testing rates in initial stages and the existence of many more cases than were validated, thus explaining apparent excessively rapid growth in case numbers.
Additional NPIs included	Social distancing, Quarantine (post exposure), Isolation (post infection), Stay-at-home (voluntary), Stay-at-home (mandatory, e.g., government-ordered)
Additional data sources used	CDC disease statistics; past model calibration experience for COVID
Previous use cases	Flu and smallpox in the U.S.
References	(18–20)
Co-authors	Chrysm Watson Ross, Tim Germann, Geoffrey Fairchild, Sara Del Valle (PI)
Funding	The LANL team was partially funded by the Laboratory Directed Research Development Program at Los Alamos National Laboratory (20200698ER and 20200697ER) and was supported by the DOE Office of Science through the National Virtual Biotechnology Laboratory, a consortium of DOE national laboratories focused on response to COVID-19, with funding provided by the Coronavirus CARES Act. Los Alamos National Laboratory is operated by Triad National Security, LLC under Contract No. 89233218CNA000001 with the U.S. Department of Energy. The content is solely the responsibility of the authors and does not

	necessarily represent the official views of the sponsors. The funders had no role in study design, data collection, analysis, decision to publish, or preparation of manuscript.
**LANL2-Age Structured ODE**	
Description	Age-structured compartmental ODE model. Stochasticity is incorporated by selecting parameters randomly from uniform distributions for each run, where the parameter ranges are determined from literature.
Diagram	See (21)
Previous use cases	COVID-19 in New Mexico
References	(21, 22)
Co-authors	Rosalyn Cherie Rael, Julie Spencer, Isabel Crooker, Carrie Manore (PI)
Funding	The LANL team was partially funded by the Laboratory Directed Research Development Program at Los Alamos National Laboratory (20200698ER and 20200697ER) and was supported by the DOE Office of Science through the National Virtual Biotechnology Laboratory, a consortium of DOE national laboratories focused on response to COVID-19, with funding provided by the Coronavirus CARES Act. Los Alamos National Laboratory is operated by Triad National Security, LLC under Contract No. 89233218CNA000001 with the U.S. Department of Energy. The content is solely the responsibility of the authors and does not necessarily represent the official views of the sponsors. The funders had no role in study design, data collection, analysis, decision to publish, or preparation of manuscript. The publication is approved for release LA-UR-20-27777.
**MESALab-FOSP**	
Description	Integer order generalized SEIR compartmental models with power law infection rates and age structure
References	(23)
Co-authors	Lihong Guo, Yanting Zhao, YangQuan Chen (PI)
**MESALab-FOSP2**	
Description	Fractional order generalized SEIR compartmental models with power law infection rates
References	(23)
Co-authors	Lihong Guo, Yanting Zhao, YangQuan Chen (PI)
**NEU-MOBS**	
Description	Stochastic, age-structured, compartmental model, including symptomatic and asymptomatic transmissions, as well as hospitalizations.
Calibration	Calibration of R0 and initial date performed using reported deaths
Additional NPIs included	Social distancing, Stay-at-home (mandatory, e.g., government-ordered)
Additional data sources used	Age structure contact patterns from highly detailed macro (census) and micro (survey) data on key socio-demographic features
References	(24)
Co-authors	Kunpeng Mu, Ana Pastore y Piontti, Alessandro Vespignani (PI)
Acknowledgements	Matteo Chinazzi, Jessica T. Davis, Xinyue Xiong
Funding	AV, APyP, KM acknowledge the support of the McGovern Foundation, Google Cloud and Google Cloud Research Credits program.
**NIH-FDA SICR**	

Description	Compartmental model with compartments for Susceptible, Infected, Case (C), case Recovered (R), and case Dead (D). The mean dynamics of the compartments are governed by an ODE system. The likelihood for the rate of appearance of C, R, and D are given by a negative binomial distribution where the dispersion parameter is a fitted parameter. Code available: https://github.com/ccc1685/covid-19
Diagram	See Fig. 1 in Chow et al.[48]
Calibration	Priors were obtained from posteriors of fits to New York and Maryland
Previous use cases	COVID-19 globally, data permitting
References	(25)
Co-authors	Joshua C Chang, Richard C Gerkin, Shashaank Vattikuti, Artur Belov, Osman Yogurtcu, Carson C Chow (PI)
Acknowledgements	Hong Yang
Funding	CCC and SV were supported by the Intramural Program of the NIH, NIDDK. RCG was supported by NIDCD, NINDS, and NSF. This work utilized the computational resources of the NIH HPC Biowulf cluster (http://hpc.nih.gov).
**Notre Dame-FRED**	
Description	Agent-based model, FRED (Framework for Reconstructing Epidemic Dynamics), with updated epidemiological parameters based on studies to date. FRED explicitly models transmission dynamics of a pathogen in a realistic population, and allows for the impacts of NPIs to be modeled explicitly (e.g., school closure results in agents representing children staying home). Code available: https://github.com/confunguido/covid19_ND_forecasting
Calibration	Disease specific parameters were calibrated to the number of daily deaths in PA. Adams County was then simulated to estimate the rate of importations from the state incidence and a scaling factor to google mobility trends. Parameters were uniformly sampled for each step of the calibration using a sobol design sequence (pomp package in R). Then, the likelihood based on the daily number of deaths was calculated.
Additional NPIs included	Isolation (post infection)
Additional data sources used	NY times data to match the daily deaths of the state of PA as a pre-fitting step (https://github.com/nytimes/covid-19-data); Google mobility trends
Previous use cases	Several diseases; originally developed by University of Pittsburgh to model the 2009 influenza pandemic
References	(26)
Co-authors	Guido España, Sean Cavany, Rachel Oidtman, T. Alex Perkins (PI)
Acknowledgements	Alan Costello, Annaliese Wieler, Anita Lerch, Carly Barbera, Marya Poterek, Quan Tran
Funding	This work was supported by an NSF RAPID grant to TAP (DEB 2027718), an Arthur J. Schmitt Fellowship and Eck Institute for Global Health Fellowship to RJO. We thank the University of Notre Dame Center for Research Computing for computing resources.
**UCLA-SuEIR**	
Description	New epidemic compartmental model (SuEIR) based on the standard SEIR model that also takes into account untested/unreported cases. The model is trained by machine learning algorithms based on reported historical data. Project website: https://covid19.uclaml.org/
Diagram	See Fig. 1 in (27)

Additional NPIs included	Quarantine (post exposure), Isolation (post infection), Stay-at-home (voluntary), Stay-at-home (mandatory, e.g., government-ordered), Age-specific interventions (e.g., isolation of elderly)
References	(27)
Co-authors	Difan Zou, Weitong Zhang, Lingxiao Wang, Pan Xu, Jinghui Chen, Quanquan Gu (PI)
**UF COVID-ABM**	
Description	Spatially explicit, agent-based model simulating a community of individuals based on census, workplace, and school data. The movement of each person during a simulated day takes place among a set of pre-assigned local places. Pathogen exposure events occur probabilistically when a susceptible person co-localizes with an infectious person and exposures can be resisted, or result in asymptomatic, mild, severe and/or critical infection
Additional NPIs included	Social distancing, Stay-at-home (voluntary), School closures
Additional data sources used	The American Community Survey 5-year dataset; geographical coordinates and the business type from the National Corporation Directory; North American Industry Classification System to identify essential vs non-essential businesses; University of Florida GeoPlan Center shapefile and data from the National Center for Education Statistics to locate schools
Previous use cases	Dengue in Yucatan, Mexico
References	(28–30)
Co-authors	Kok Ben Toh, Arlin Stoltzfus, Carl Pearson, Dianela Perdomo, Alexander Pillai, Sanjana Bhargava, Thomas Hladish (PI)
	CP acknowledges the Bill & Melinda Gates Foundation (OPP1184344) and the UK Foreign, Commonwealth and Development Office (FCDO)/Wellcome Trust Epidemic Preparedness Coronavirus research programme (ref. 221303/Z/20/Z)
**UNCC LSTM**	
Description	Data-driven, stochastic SI model utilizing a deep learning recurrent neural network with multivariate LSTM architecture. The model was calibrated using COVID-19 epidemic data in another region with ending of the epidemic to guide the model to learn how the epidemic could eventually phase out.
Calibration	Transfer learning was used to let the LSTM learn how the epidemic would eventually end from another region, explore the RNN structure and hyperparameters, and apply them to tune the model for the modeled region
Additional data sources used	COVID-19 data from another region where the epidemic has (presumably) ended.
Co-authors	Daniel Janies, Rajib Paul, Shi Chen (PI)
Acknowledgements	Tinghao Feng
**UT-SEPAYHR**	
Description	Stochastic, age- and risk-structured compartmental model that includes susceptible, exposed, presymptomatic, asymptomatic, symptomatic, hospitalized, and recovered states (SEPAYHR). The model is simulated using a hybrid approach with a deterministic initial phase (up to 20 total symptomatic cases) followed by a stochastic phase.
Diagram	See Fig. A1 in (31)
Calibration	Basic reproductive number (R_t) was estimated using provided and transmission probability was estimated using a next-generation matrix approach based on the model structure and R_t. Epidemic start date was based on the time to first death implied by the estimated Rt and transmission probability. Transmission reduction due to social distancing was estimated with a nonlinear least

	squares fitting procedure in the SciPy/Python package. Detection rate was estimated using the provided data and published estimates of age-structured infection fatality ratios.
Additional NPIs included	Social distancing, Stay-at-home (voluntary), Stay-at-home (mandatory, e.g., government-ordered)
References	(31)
Co-authors	Kelly Pierce, Remy Pasco, Lauren Ancel Meyers (PI)
Acknowledgements	Spencer Fox, Zhanwei Du, Ethan Ho, Greg Zynda, Jawon Song
Funding	CDC contract 75D-301-19-C-05930, and NIH grant 3R01AI151176-01S1
**UW-THINKLAB-SEIQRD**	
Description	Compartmental model, consisting of 6 compartments: Susceptible (S), Exposed (E), Infectious (I), Quarantined (Q), Recovered (R) and Dead (D). Transitions between compartments are formulated using deterministic functions in discrete time steps and parameters governing transitions are assumed to change stochastically on a daily basis (except for predetermined parameters).
Calibration	Particle filtering is used to update the distribution of parameter estimates on a daily basis while case and death data is available (i.e. by May 15).
Additional data sources used	National average hospital beds per capita from World Health Organization (https://www.who.int/data/gho/data/indicators/indicator-details/GHO/hospital-beds-(per-10-000-population)
Co-authors	Xiangyang Guan, Cynthia Chen (PI)
**VT Childs Lab**	
Description	Deterministic, compartmental ODE system. Parameter sets are chosen using Latin Hypercube Sampling and refined based on comparison to data.
Calibration	Parameters were chosen from given ranges via Latin Hypercube Sampling (LHS)
Additional NPIs included	Social distancing, Isolation (post hospitalization)
Co-authors	Lauren M Childs (PI)
Acknowledgements	Kate Langwig, Leah Johnson, Eyvindur Ari Palsson, Julie Blackwood
Funding	LMC acknowledges support from National Science Foundation grant No. 2029262.

Table S2. Importation rate. Most models did not include an importation rate after any initial seeding. Models that did maintained a relatively small importation rate, per the elicitation setting.

Model ID	Importation rate (or None)
A	None
B	None
C	None
D	None
E	0.14 cases / day
F	1 exposure / day (with a probability of resistance)
G.1	None
G.2	None
H	None
I	None (after initial seeding)
J	None (after initial seeding)
K	None
L	0 – 0.5 cases / day
M	None
N	None
O	Varied
P	None

The original project description and request for contributions to MMODS Elicitation 1 is appended to this supplement. The request includes a detailed description of the MMODS process as well as information on the setting, interventions, and objectives to be considered during the first MMODS collaboration exercise.

The submission form and checklist of data required for round 2 models is appended to this supplement. It comprised a detailed web form requiring the reporting of information characterizing the model being submitted. This form was hosted behind a login portal for participating model groups on the MIDAS website.

Movie S1. MMODS_Elicitation1_InterventionRankResults.mp4
Model-specific intervention rank results evaluated for each objective in round 2 of the MMODS process. Results are displayed in video format by quantile (1 through 100). Colors indicating ranks and rank-ties are as specified for the median rank result figures in main text and range from single best intervention (dark blue) to single worst intervention (dark red).

Data S1. MMODS_Elicitation1-ProvidedData.xlsx
Data for a generic U.S. county were provided to modeling groups to inform their model specifications. Provided data include: epidemiological data on daily and cumulative cases and deaths from January 22 to May 15, 2020; demographic information on age- and sex-distribution; testing and mobility; timing on the release of a stay-at-home order and a State of Emergency declaration. Data adapted from (9–11, 13, 32, 33).

Data S2. MMODS_Elicitation1-OutputData. xlsx
Anonymized results for individual models and the aggregate. Results are provided in a standard format, including 100 quantiles for each model-objective-intervention (or aggregate-objective-intervention) combination (i.e., the probability distribution for each outcome for each intervention, via the cumulative distribution function (CDF) in 100 quantiles).

References and Notes

1. P. E. Tetlock, B. A. Mellers, N. Rohrbaugh, E. Chen, Forecasting Tournaments: Tools for Increasing Transparency and Improving the Quality of Debate. *Curr Dir Psychol Sci* **23**, 290–295 (2014).

2. S. den Boon, *et al.*, Guidelines for Multi-Model Comparisons of the Impact of Infectious Disease Interventions. *BMC Medicine* **17**, 163 (2019).

3. M. K. Murphy, *et al.*, Consensus Development Methods, and Their Use in Clinical Guideline Development. *Health Technology Assessment (Winchester, England)* **2**, i–iv, 1–88 (1998).

4. M. A. Burgman, *Trusting judgements: how to get the best out of experts* (Cambridge University Press, 2015).

5. N. Mukherjee, *et al.*, The Delphi Technique in Ecology and Biological Conservation: Applications and Guidelines. *Methods in Ecology and Evolution* **6**, 1097–1109 (2015).

6. P. E. Tetlock, D. Gardner, *Superforecasting: The Art and Science of Prediction* (Random House, 2016).

7. S.-L. Li, *et al.*, Concurrent assessment of epidemiological and operational uncertainties for optimal outbreak control: Ebola as a case study. *Proceedings of the Royal Society B: Biological Sciences* **286**, 20190774 (2019).

8. E. L. Ray, *et al.*, Ensemble Forecasts of Coronavirus Disease 2019 (COVID-19) in the U.S. *medRxiv*, 2020.08.19.20177493 (2020).

9. E. Dong, H. Du, L. Gardner, An Interactive Web-Based Dashboard to Track COVID-19 in Real Time. *The Lancet Infectious Diseases* **20**, 533–534 (2020).

10. B. D. Killeen, *et al.*, A County-Level Dataset for Informing the United States' Response to COVID-19. *arXiv:2004.00756 [physics, q-bio]* (2020).

11. Keystone Strategy, COVID-19 Intervention Data (August 10, 2020). https://github.com/Keystone- Strategy/covid19-intervention-data/.

12. NACO, Counties and COVID-19 Safer at Home Orders (August 10, 2020). https://www.naco.org/resources/featured/counties-and-covid-19-safer-home-orders.

13. Google COVID-19 Community Mobility Reports (August 10, 2020). https://www.google.com/covid19/mobility/.

14. Georgetown University Center for Global Health Science and Security, Talus Analytics, Nuclear Threat Initiative (NTI), COVID Act Now, COVID Analysis and Mapping of Policies (AMP) https://covidamp.org/. (November 15, 2020).

15. *COVID-19 App V15.1.3* (2020).

16. C. C. Kerr, *et al.*, Covasim: An Agent-Based Model of COVID-19 Dynamics and Interventions. *medRxiv*, 2020.05.10.20097469 (2020).

17. G. Lin, *et al.*, Explaining the Bomb-Like Dynamics of COVID-19 with Modeling and the Implications for Policy. *medRxiv*, 2020.04.05.20054338 (2020).

18. T. C. Germann, K. Kadau, I. M. Longini, C. A. Macken, Mitigation Strategies for Pandemic Influenza in the United States. *Proceedings of the National Academy of Sciences* **103**, 5935–5940 (2006).

19. M. E. Halloran, *et al.*, Modeling Targeted Layered Containment of an Influenza Pandemic in the United States. *Proceedings of the National Academy of Sciences* **105**, 4639–4644 (2008).

20. T. C. Germann, *et al.*, School Dismissal as a Pandemic Influenza Response: When, Where and for How Long? *Epidemics* **28**, 100348 (2019).

21. J. Spencer, "Chronic and Acute Respiratory Pathogens: Evolutionary and Epidemiological Characteristics of Tuberculosis, Influenza-like Illness, and COVID-19.," University of New Mexico, Albuquerque, New Mexico. (2020).

22. J. Spencer, *et al.*, Epidemiological Parameter Review and Comparative Dynamics of Influenza, Respiratory Syncytial Virus, Rhinovirus, Human Coronavirus, and Adenovirus. *medRxiv*, 2020.02.04.20020404 (2020).

23. L. Guo, Y. Zhao, Y. Chen, Management Strategies and Prediction of COVID-19 by a Fractional Order Generalized SEIR Model. *medRxiv*, 2020.06.18.20134916 (2020).

24. D. Mistry, *et al.*, Inferring High-Resolution Human Mixing Patterns for Disease Modeling. *arXiv:2003.01214 [physics, q-bio]* (2020).

25. C. C. Chow, J. C. Chang, R. C. Gerkin, S. Vattikuti, Global Prediction of Unreported SARS-CoV2 Infection from Observed COVID-19 Cases. *medRxiv*, 2020.04.29.20083485 (2020).

26. J. J. Grefenstette, *et al.*, FRED (A Framework for Reconstructing Epidemic Dynamics): An Open-Source Software System for Modeling Infectious Diseases and Control Strategies Using Census-Based Populations. *BMC Public Health* **13**, 940 (2013).

27. D. Zou, *et al.*, Epidemic Model Guided Machine Learning for COVID-19 Forecasts in the United States. *medRxiv*, 2020.05.24.20111989 (2020).

28. T. J. Hladish, *et al.*, Designing Effective Control of Dengue with Combined Interventions. *Proceedings of the National Academy of Sciences* **117**, 3319–3325 (2020).

29. T. J. Hladish, *et al.*, Forecasting the Effectiveness of Indoor Residual Spraying for Reducing Dengue Burden. *PLOS Neglected Tropical Diseases* **12**, e0006570 (2018).

30. S. Flasche, *et al.*, The Long-Term Safety, Public Health Impact, and Cost-Effectiveness of Routine Vaccination with a Recombinant, Live-Attenuated Dengue Vaccine (Dengvaxia): A Model Comparison Study. *PLoS medicine* **13**, e1002181 (2016).

31. D. Duque, *et al.*, *Staged Strategy to Avoid Hospital Surge and Preventable Mortality, While Reducing the Economic Burden of Social Distancing Measures* (2020).

32. Pennsylvania Department of Health, COVID-19 Data for Pennsylvania (2020) https://www.health.pa.gov/topics/disease/coronavirus/Pages/Coronavirus.aspx (May 27, 2020).

33. U.S. Census Bureau, Current Population Survey, 2019 Annual Social and Economic Supplement (2019) https://www.census.gov/data/datasets/2019/demo/cps/cps-asec-2019.html (April 30, 2020).

Harnessing Multiple Models for Outbreak Management
Exercise I. Relaxation of social distancing

The Problem

The profusion of models for COVID-19, with differing structures, varied epidemiological scenarios, parameters and presentation, and sometimes conflicting projections, is a challenge for decision-makers. In a recent paper, we proposed a method for harnessing the power of multiple models by drawing from tools in decision analysis, expert judgment, and model aggregation (Shea et al. 2020). This project is meant to implement that proposal in the context of COVID-19. We aim to generate *unbiased and well-calibrated aggregate projections under different interventions,* that encapsulate scientific and logistical uncertainty, to better inform management decisions. In this framework, insights can be shared across groups to inform the same decision, while retaining the perspective of individual groups as part of the full expression of uncertainty.

The overall goals of this project are to implement these procedures for a series of COVID-19 decisions; engage a diverse set of modeling groups with expertise in structured, collaborative, ensemble projections; and develop efficient logistical processes for managing our broad communal effort. This is a complementary effort to the COVID-19 forecasting hub developed by Nick Reich and colleagues, with an explicit focus on interventions and decisions.

Specifically, we will run multiple projection exercises to address key decisions facing managers of COVID-19, including when and how to relax key social distancing interventions (exercise I). In later exercises, we will use model assemblages to assess more nuanced partial reopening strategies, intervention decisions at state and country levels, where best to trial vaccines and drugs, how to prioritize testing and how to optimize the roll-out of medical interventions. We will request, from each participating group, one or more models that encapsulate their group's best understanding of the current pandemic (that is, we will treat each model as an hypothesis about the current outbreak). All participants will be invited as co-authors on resultant publications. Results will be kept confidential within the group until presentation to decision-makers or in publication(s). When presented outside the group, only model participation will be disclosed (individual model results will be anonymized).

Procedure

We will use principles of decision analysis to help structure model projections and analysis, and adopt well-established methods from the expert judgment literature so that the results from multiple modeling groups can all contribute to insights about the same decision context and contribute to a synthetic and long term resolution to the current pandemic.

For each exercise, we will take the following steps:

1. *Setting*. We will present a decision setting, specifying the background epidemiology (location, outbreak trajectory), the targets of the decision maker (e.g., minimizing deaths, epidemic duration, etc.), and the intervention(s) to examine. Relevant epidemiologic and demographic data will be shared.
2. *Individual Projections 1*. We will ask each modeling group to independently estimate the

desired outcomes under the alternative interventions, with particular attention to expressing uncertainty. We will ask for probability distributions for each outcome and intervention scenario.

3. *Group Discussion*. We will compile the results from the multiple modeling groups and display them (anonymously) in a format that permits ready comparison. We will convene a group discussion with all the modeling groups to explore the commonalities and differences, to share insights, and to discuss sources of uncertainty.
4. *Individual Projections 2*. We will then ask each modeling group to independently project the same targets under the alternative interventions again, taking into account the insights from the group discussion to the extent they find them compelling. We will again ask for probability distributions.
5. *Aggregation and Analysis*. We will then aggregate the second round of results into a set of ensemble projections that captures the uncertainty within and across modeling groups. We will also conduct a value-of-information analysis to identify sources of uncertainty that most affect the choice of an intervention. The summary of this work should be an analysis that conveys to the decision maker the expected performance of each of the interventions, using the ensemble projections, with an understanding of the role of uncertainty.

Exercise I of the elicitation: post-lockdown strategies

Setting and initial conditions:

We ask that you consider the setting of a US county of 100,000 people, with an age structure typical of the age structure across the US, that pre-emptively initiated, and adhered to, stringent social distancing guidelines (i.e., full lockdown with workplace and school closures) until May 15th, 2020. As of 15th May 2020, the town has recorded 180 confirmed cumulative cases and 6 total deaths (time series for both provided). Please assume current (i.e., partial) travel restrictions remain in place throughout the exercise, so that no international importation is allowed and domestic importations are limited.

The decision maker is the county executive, who has authority to specify guidance for opening workplaces. The focus is on decisions regarding social distancing and re-opening over the next few months, prior to the onset of the influenza season.

Projection outcomes/objectives:

The county executive has indicated they are interested in weighing the trade-offs among a number of outcomes, including the impact of the disease on public health, hospital resources, and the local economy. To reflect these objectives, we ask participating modeling groups to address **5** outcomes (metrics):

1) cumulative number of infected individuals through November 15
2) cumulative number of deaths through November 15
3) peak hospitalizations through November 15
4) probability of a new local outbreak (more than 10 new cases/day) before November 15
5) total number of days workplaces closed through November 15

Interventions
In this first exercise, we will only consider relaxation related to workplaces. We request that you provide model projections for the following **4** intervention scenarios:

1) continue with current workplace and school closures until November 15
   (baseline full control scenario)

2) relax current social distancing 2 weeks after peak:
   - open workplaces only (schools remain closed through November 15)

3) relax social distancing when the number of new daily cases is at 1% of peak
   - open workplaces only (schools remain closed through November 15)

4) immediately relax all current restrictions on workplaces
   (schools remain closed through November 15)

For now, please assume no local testing/contact tracing and isolation of infected individuals; we will return to evaluate this in a future elicitation. You are however free to define and present results for any other relaxation process you feel is relevant or interesting.

Models should provide a full probability distribution of outcomes for each intervention, such that tail probabilities for the 2nd and 98th quantiles are relatively stable. Specifically, we want the probability distribution for each outcome for each intervention, by specifying the cumulative distribution function (i.e., with 100 quantiles). We will provide a submission template.

We request submissions by 9 June 2020. Please provide your contact information in the Google Spreadsheet if you plan to participate, and we will give you more detailed submission information. In case of questions, email Dr. Katriona Shea (k-shea@psu.edu).

Background information on your model: Please provide a short write up of your model, including assumptions made about key epidemiological parameters, with parametric uncertainty (e.g., transmission, recovery, R_0, serial interval). Please document all sources of variation in your model using the checklist provided. We are looking for full expression of uncertainty in these projections. For example, uncertainty may be structural (e.g., should asymptomatic carriers be modeled explicitly?), or parametric with respect to the biology (e.g., what is the expected time between sequential cases in a chain of transmission?), the setting (what is the assumed rate of domestic importations?) or the interventions (e.g., what is the expected impact of social distancing?) or there may be other sources of stochasticity. Other key uncertainties you might scan across might include: controllability of social distancing, probability of novel incursions that might lead to a second wave of local infections, etc. Details of any model calibration or inference framework used should be provided (checklist will be provided). If we do not specify something, please use your best judgment and include that in your modeling of uncertainty (and please let us know in your short model description and in the checklist). Do not hesitate to send questions, and please provide any other information you feel is pertinent so we can update our checklist for future exercises.

Multi-Model Outbreak Decision Support (MMODS)

Submission Form

MMODS Homepage
Change Password
Logout

You are submitting as:
loginEmail

Models you've submitted already:
None.

Save your work, or load work that you've saved!

Load answers from a previously saved or submitted model:

Load Clear Answers

Save Progress Note: Submission Files aren't saved as they shouldn't be re-used.

Please be sure to save your work by clicking the "Save Progress" button. Any unsaved work will be lost if you refresh or leave this page. Submissions will be automatically saved upon successful submission.

All fields marked * are required for submission.

Please be sure that your submission file meets the requirements specified in the submission template, and upload your file here. In order for your submission to be considered, please fill out the form below and then click the submit button when finished (allowed filetypes are csv, xlsx, and xls):

Choose File no file selected

Model Short Name (please limit to 15 characters or fewer):*

Model Description (please be sure to tick all that apply):*

- ☐ Deterministic
- ☐ Individual-based (agent-based) model
- ☐ Spatially explicit
- ☐ Stochastic
- ☐ Compartmental Model
- ☐ Other

Please provide a brief model description, including an explanation of the ways in which the above selected description(s) are applicable (e.g., to different parts or parameters of your model). Note, if you would like to include a model diagram, please do so as an optional file upload at the end*:

Model Component (e.g. compartment, probability, or rate)

Susceptibility*:

Type (or lack) of symptoms*:

Disease severity (e.g. mild/severe)*:

Time until infectious*:

Time until symptomatic*:

Reinfection (e.g. waning immunity)*:

Hospitalization (of any kind)*:

Entering intensive care/treatment unit (ICU/ITU)*:

Recovery*:

Disease related death/mortality*:

Testing*:

If applicable, indicate age groups used (if multiple age groups are used in different components of the model, please detail):

Please describe any other model components or structures considered (e.g. health conditions, pathogens besides SARS-CoV-2 simultaneously modelled, other groups/stratifications included):

MODEL PARAMETERS, UNCERTAINTY, AND OUTCOMES

Please enter the distribution of values (5th, 25th, 50th, 75th, 95th quantiles) considered for the initial number of susceptible individuals (i.e., on May 15). If a distribution is not possible, then please provide a single median value::*

Please indicate importation rate(s) used (e.g. 1 case / week), if included in the model:

Please detail any assumed relationships between infected numbers and reported cases (e.g., reporting rate(s) used):*

Please detail modeling choices regarding proportion of asymptomatic individuals:*

If your model is able to produce a probability distribution for the probability of an outbreak, please provide the full distribution in the submission template and detail methods used to produce the distribution here:

Please enter the distribution of dates (5th, 25th, 50th, 75th, 95th quantiles) at which the 5% intervention was set in motion (if a distribution is not possible, then please provide a single median value, or if not attained, indicate NA):*

Please enter the distribution of dates (5th, 25th, 50th, 75th, 95th quantiles) at which the 2-week intervention was set in motion (if a distribution is not possible, then please provide a single median value, or if not attained, indicate NA):*

Please enter the distribution of values (5th, 25th,50th, 75th, 95th quantiles) observed for the final number of susceptible individuals (i.e., on Nov 15). If a distribution is not possible, then please provide a single median value:*

If available, please provide the distribution of values (5th, 25th,50th, 75th, 95th quantiles) observed for the daily new infections on final day (i.e., on Nov 15th). If a distribution is not possible, then please provide a single median value:

If available, please provide the distribution of values (5th, 25th,50th, 75th, 95th quantiles) observed for the daily deaths on final day (i.e., on Nov 15th). If a distribution is not possible, then please provide a single median value:

Below, please indicate whether component value(s) are fixed or whether uncertainty was accounted for and if so, how. . For each component, select the option that applies best from the following:

- N/A (component not included)
- Fixed (no uncertainty expressed regarding this component)
- Uncertainty - Likelihood-based (uncertainty included; estimated using likelihood-based methods)
- Uncertainty – Simulation (uncertainty included; values explored through simulation)
- Uncertainty - Expert Judgment (uncertainty included; estimated using expert judgment), or
- Uncertainty – Other (uncertainty included; values estimated by methods other than above)

Model Component

Susceptibility:*

Type (or lack) of symptoms:*

Disease severity (e.g. mild/severe):*

Time until infectious:*

Time until symptomatic:*

Reinfection (e.g. waning immunity):*

Hospitalization (of any kind):*

Entering intensive care/treatment unit (ICU/ITU):*

Recovery:*

Disease related death/mortality:*

Testing:*

Please provide any additional details you think are important to note regarding uncertainty about model components here:

Please describe methods used to address any structural or other sources of uncertainty in your model, if applicable:

Interventions

- ☐ Tick if interventions aside from non-essential workplace closures were explicitly included in the model.

Please tick boxes next to any additional interventions that were incorporated in your Elicitation 1 model here:

- ☐ Contact tracing (digital)
- ☐ Contact tracing (manual)
- ☐ Social distancing
- ☐ Quarantine (post exposure)
- ☐ Isolation (post infection)
- ☐ Stay-at-home (voluntary)
- ☐ Stay-at-home (mandatory, e.g., government-ordered)
- ☐ Age-specific interventions (e.g., isolation of elderly)

Other(s):

Please detail model assumptions regarding compliance with NPIs, including for non-essential workplace closures:

Pharmaceutical interventions (PIs):

- ☐ Testing (for active infection, e.g. PCR testing)
- ☐ Testing (for past infection, e.g. antibody testing)

If testing was included:

Please detail assumptions regarding testing location (i.e. community testing):

Please detail assumptions regarding testing rate:

Please list any other intervention(s) modeled and related assumptions:

DATA

Please tick boxes next to all data sources provided as part of this elicitation that were used:*

- ☐ Cases
- ☐ Deaths
- ☐ Mobility
- ☐ Demographic
- ☐ Testing
- ☐ Stay at home / State of emergency
- ☐ None of the above data provided as part of this elicitation were used

Please describe/provide links to any additional data sources used:

MODEL CALIBRATION

- ☐ Model calibration was performed (that is, some aspects of parameter estimation were conducted using the model structure and the available data).

MODEL HISTORY

- ☐ Model has been used before now.

If research using this model has been published, tick all publication types in which the model has appeared that apply:

- ☐ White paper
- ☐ Peer-reviewed journal
- ☐ Pre-print server

If model has been used (in published research) before being applied to COVID-19, please detail other pathogen(s) to which the model has been applied and/or which locations (e.g. region, country) where this model has been previously applied:

Please include links/references to any key publications or pre-prints where the model has been used previously:

ADMINISTRATION

How many person-hours do you estimate were allocated to this modeling effort?*

ELICITATION

Please provide any feedback or suggestions as to what you think should have been requested for this and/or future elicitations (e.g. other objectives/metrics, interventions, settings, sources of uncertainty, or additional pertinent information that should have been explicitly requested):

To help with future elicitations, please provide any comments regarding this process that would have made it easier on participants:

ADDITIONAL INFORMATION

If you have any additional documentation files available, that you are willing to share, please upload them here (e.g., epidemic curves, distributions of the underlying parameters, model diagram, or manuscript describing the model). Note there is a 5MB upload limit. Allowed file types are: csv, xls, xlsx, pdf, jpg, and png. Select multiple files in your browser window if you'd like to upload multiple files:

Choose Files no files selected

Please provide any other general comments or suggestions here:

Submit

The content of this page is confidential and research in progress. Please do not share or cite.

MIDAS
MIDAS Coordination Center
University of Pittsburgh

A737 Public Health
130 DeSoto Street
Pittsburgh PA 15261

COPYRIGHT © MIDAS 2020
